# MBD5 and MBD6 stabilize the BAP1 complex and promote BAP1-dependent cancer

**DOI:** 10.1186/s13059-022-02776-x

**Published:** 2022-09-30

**Authors:** Natsumi Tsuboyama, Aileen Patricia Szczepanski, Zibo Zhao, Lu Wang

**Affiliations:** 1 Department of Biochemistry and Molecular Genetics, Feinberg School of Medicine, Northwestern University, Chicago, IL 60611 USA; 2 Simpson Querrey Center for Epigenetics, Feinberg School of Medicine, Northwestern University, SQBRC 7-404, 303 E. Superior St., Chicago, IL 60611 USA; 3 Robert H. Lurie Comprehensive Cancer Center, Northwestern University Feinberg School of Medicine, Chicago, IL 60611 USA

**Keywords:** MBD5, MBD6, The BAP1 complex, PHD fingers, SCLC

## Abstract

**Background:**

BRCA1-associated protein 1 (BAP1) is an ubiquitin carboxy-terminal hydrolase, which forms a multi-protein complex with different epigenetic factors, such as ASXL1-3 and FOXK1/2. At the chromatin level, BAP1 catalyzes the removal of mono-ubiquitination on histone H2AK119 in collaboration with other subunits within the complex and functions as a transcriptional activator in mammalian cells. However, the crosstalk between different subunits and how these subunits impact BAP1’s function remains unclear.

**Results:**

We report the identification of the methyl-CpG-binding domain proteins 5 and 6 (MBD5 and MBD6) that bind to the C-terminal PHD fingers of the large scaffold subunits ASXL1-3 and stabilize the BAP1 complex at the chromatin. We further identify a novel Drosophila protein, the six-banded (SBA), as an ortholog of human MBD5 and MBD6, and demonstrate that the core modules of the BAP1 complex is structurally and functionally conserved from Drosophila (Calypso/ASX/SBA) to human cells (BAP1/ASXL/MBD). Dysfunction of the BAP1 complex induced by the misregulation/mutations in its subunit(s) are frequent in many human cancers. In BAP1-dependent human cancers, such as small cell lung cancer (SCLC), MBD6 tends to be a part of the predominant complex formed. Therefore, depletion of MBD6 leads to a global loss of BAP1 occupancy at the chromatin, resulting in a reduction of BAP1-dependent gene expression and tumor growth in vitro and in vivo.

**Conclusions:**

We characterize MBD5 and MBD6 as important regulators of the BAP1 complex and maintain its transcriptional landscape, shedding light on the therapeutic potential of targeting MBD5 and MBD6 in BAP1-dependent human cancers.

**Supplementary Information:**

The online version contains supplementary material available at 10.1186/s13059-022-02776-x.

## Background

Systematic genomic analysis has revealed that many epigenetic factors are aberrantly expressed or somatically mutated in cancer [1, 2]. Therefore, it is expected that understanding the mechanisms of epigenetic regulation in cancer cells will lead to the elucidation of the regulatory mechanisms involved in cancer development and progression, and eventually to the discovery of effective diagnostic and therapeutic targets [3–5].

BRCA1-associated protein 1 (BAP1) was first identified as a deubiquitinating enzyme that binds to the BRCA1-Ring finger [6]. Later, in *Drosophila*, Calypso (an ortholog of BAP1) was shown to form a complex with additional sex combs (ASX) to remove mono-ubiquitin from histone H2A [7]. It has been further demonstrated that the Calypso/ASX heterodimer binds to PcG target genes and is critical for the repression of Hox gene expression in *Drosophila* cells [7].

In mammalian cells, BAP1 functions as a more complicated multi-protein complex, which contains as many as ten different subunits, including the additional sex comb-like protein 1-3 (ASXL1-3), FOXK1/2, OGT, and HCTC1 [8–12]. These subunits are found to be involved in controlling the stability of the complex [9], deubiquitinase activity [13], and chromatin recruitment [8, 14, 15]. For instance, the ASXL1-3 proteins, known as being the largest subunits within the BAP1 complex, can bind to the C-terminus of BAP1 in a mutually exclusive manner via their N-terminal domains, which are critical for activation of BAP1’s catalytic activity towards the removal of H2AK119Ub [13]. Recent studies have revealed that the chromatin binding of BAP1 depends on FOXK1/2 subunits [8]. Nevertheless, FOXK1/2 are also important components within other epigenetic complexes, such as the Sin3A complex. FOXK1/2 depletion leads to a site-specific loss of BAP1’s association with chromatin, and a subsequent repression of gene expression [8].

Consequently, abnormalities in these subunits may affect their executive function and assembly as a complex and influence the development and progression of cancer. Our previous studies have shown that leukemia-specific C-terminus truncated ASXL1 is able to stabilize BAP1 and enhance BAP1’s recruitment to chromatin which promotes the expression of a pro-leukemic transcriptional signature [16]. Moreover, we have recently characterized a human small cell lung cancer (SCLC)-specific epigenetic axis comprised of BAP1/ASXL3/BRD4 [15]. In SCLC cells, the lineage-specific scaffold subunit ASXL3 directly links BRD4 to BAP1 at active enhancers and drives oncogenic gene expression [17]. Genetic depletion of BAP1 or inhibition of BAP1 activity by small molecule inhibitors [18] could dramatically reduce the tumor growth of BAP1-dependent cancer, such as ASXL1-mutant leukemia [19], breast cancer [19], and SCLC [18].

Among the potential BAP1-associated factors, the methyl-CpG-binding domain proteins 5 and 6 (MBD5 and MBD6) have been previously shown to interact with a few of the components within the BAP1 complex by in vitro protein purification, with BAP1 [10, 20] or ASXL1/2 [21] as the bait protein. However, the specific binding site and the function of MBD5 and MBD6 in the complex remain unknown. Therefore, MBD5 and MBD6 have been excluded from the BAP1 complex due to a lack of evidence on endogenous protein-protein interaction between MBD5, MBD6, and BAP1 [12, 22–25]. In our current studies, by utilizing genome-wide studies and in vitro biochemical analysis, we have demonstrated that the endogenous MBD5 and MBD6 function as stable subunits within the BAP1 complex by binding through the MBD domain to the plant homeodomain (PHD) finger of ASXL1-3. Furthermore, depletion of ASXL subunits completely abolished the protein-protein interaction between BAP1 and MBD subunits. In addition, the MBD5 and MBD6 subunits are critical for maintaining the stability of the BAP1 complex and therefore are essential for BAP1-dependent tumor cell growth in vitro and in vivo.

## Results

### MBD5 and MBD6 are stable subunits of the BAP1 complex at chromatin

**Fig. 1:**
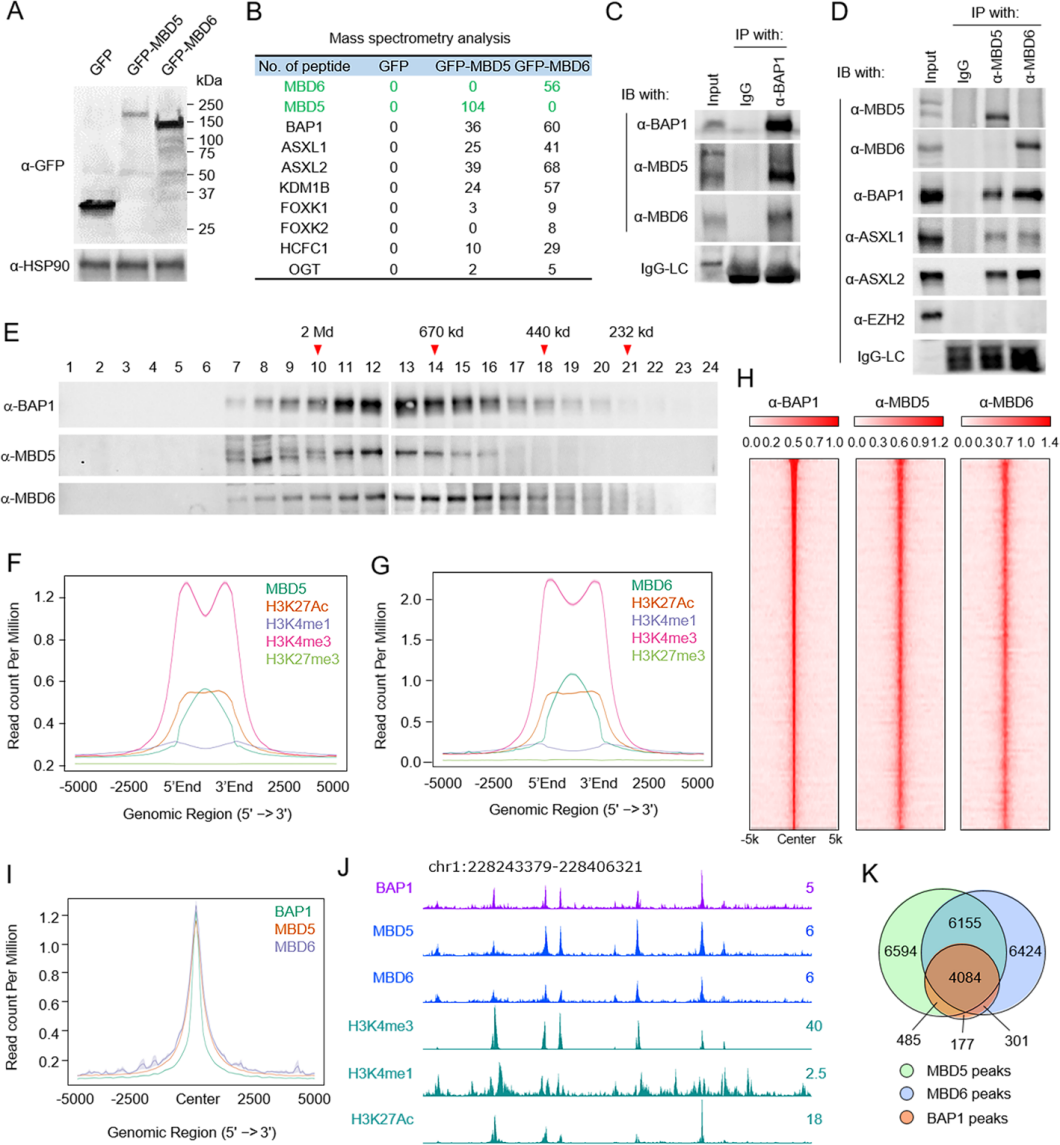
Endogenous MBD5 and MBD6 are stable components in the BAP1 complex. **A** HEK293T cells were infected by lentivirus expressing GFP, GFP-tagged MBD5, or GFP-tagged-MBD6. The protein levels of GFP, GFP-MBD5, and GFP-MBD6 were determined by western blot, *n*=2. **B** The GFP-fusion proteins were purified from HEK293T cells stably expressing GFP, GFP-MBD5, or GFP-MBD6 defined in **A**. The purified proteins were subjected to mass spectrometry analysis. Peptide numbers of subunits within the BAP1 complex pulled down by GFP-tagged proteins are shown. **C** Immunoprecipitation (IP) of endogenous BAP1 from HEK293T cells followed by immunoblot (IB) for BAP1, MBD5, and MBD6. IgG was used as a negative control, *n* = 2. **D** IP of endogenous MBD5 or MBD6 from HEK293T cells followed by IB for MBD5, MBD6, BAP1, ASXL1, and ASXL2. IgG was used as a negative control, *n* = 2. **E** Nuclear extract from HEK293T cells was subjected to size exclusion (SE) chromatography and then protein levels of BAP1, MBD5, and MBD6 were determined by western blot analysis, *n* = 2. **F** The average plots shown represent the chromatin occupancy of H3K27Ac, H3K4me1, H3K4me3, and H3K27me3 centered on MBD5 peaks (**F**) or MBD6 peaks (**G**). **H** Sorted and centered heatmaps generated from ChIP-seq data analyses show the occupancy of BAP1, MBD5, and MBD6 in HEK293T cells. All rows are centered on BAP1 peaks based on the ranking of signals. **I** The average plot shows the co-occupancy between BAP1, MBD5, and MBD6 centered on BAP1 peaks. **J** The representative tracks show H3K27Ac, H3K4me1, and H3K4me3 levels at BAP1, MBD5, and MBD6 occupied loci in HEK293T cells. **K** A Venn diagram representation of the overlapped ChIP-seq peaks between BAP1, MBD5, and MBD6

As a chromatin-bound, multi-protein complex, the BAP1 complex functions as a general transcriptional cofactor that activates transcription [21, 26, 27]. Subunits within the BAP1 complex, such as ASXL1-3 and FOXK1/2, have been demonstrated to mediate the catalytic activity or chromatin recruitment of BAP1 [8, 13]. However, the existence of the methyl-CpG binding domain protein 5 and 6 (MBD5 and MBD6, respectively) within the BAP1 complex is still an ongoing debate. Moreover, the potential role of MBD5 and MBD6 in the BAP1 complex remains to be discovered. To fully characterize the functional role of MBD5 and MBD6 in mammalian cells, we first generated GFP-tagged MBD5 and MBD6 chimeric proteins that are stably expressed in HEK293T cells (Fig. 1A). GFP-protein purification followed by mass spectrometry analysis identified the full list of subunits encompassing the BAP1 complex that co-precipitated with either MBD5 or MBD6 (Fig. 1B, Additional file 1: Table S1). To further confirm the mass spectrometry results, we individually generated polyclonal antibodies against MBD5 and MBD6 (Additional file 2: Fig. S1A), and further validated the specificity of the antibodies in MBD5 and MBD6 knockout cells (Additional file 2: Fig. S1B-D). After testing the specificity of the antibodies, we performed immunoprecipitation in HEK293T cells and demonstrated a stable protein-protein interaction between endogenous MBD5, MBD6, and BAP1 (Fig. 1C, D). Moreover, BAP1 knockout does not affect the protein levels of either MBD5 or MBD6 in cells (Additional file 2: Fig. S1E).

To further study the composition of endogenous MBD5, MBD6, and BAP1, nuclear extracts from HEK293T cells were subject to size exclusion (SE) chromatography, followed by western blot analysis of the elution profiles of MBD5, MBD6, and BAP1. As shown in Fig. 1E, we found MBD5 and MBD6 were co-eluted with BAP1. Notably, the MBD5 and MBD6 SE profiles tend to be mutually exclusive from one another, which is consistent with the mass spectrometry results in Fig. 1B and immunoprecipitation results in Fig. 1D. In summary, these results demonstrated that the endogenous MBD5 and MBD6 subunits are stable components within the BAP1 complex that form distinct complexes with BAP1. Notably, this mutually exclusive behavior of these homologous subunits within the BAP1 complex is common, as shown by ASXL1-3 and FOXK1/2.

To determine whether MBD5 and MBD6 can also bind to chromatin similar to other BAP1 subunits, we conducted Chromatin Immunopreciptiation Sequencing (ChIP-seq) and determined the chromatin binding profiles of MBD5 and MBD6 with our validated homemade antibodies (Additional file 2: Fig. S1F). As shown in Fig. 1F and G, the average plot shows a significant enrichment of histone marks (H3K4me1, H3K4me3, and H3K27Ac) at *MBD5* and *MBD6* loci. However, there are no detectable H3K27me3 levels at MBD5 or MBD6 occupied loci, which suggests that both MBD5 and MBD6 are localized at active chromatin regions. Consistent with our in vitro protein-protein interaction assay, we have also detected a significant overlap between MBD5, MBD6, and BAP1 peaks by ChIP-seq assay (Fig. 1H–J). Notably, more than 96.5% of BAP1 peaks are co-occupied by MBD5 and/or MBD6 (Fig. 1K) in HEK293T cells. In addition, by K-means clustering analysis (Additional file 2: Fig. S1G), we found that MBD5 and MBD6 occupied genes enriched in different signaling pathways, indicating that there is a functional distinction between MBD proteins (Additional file 2: Fig. S1H).

### The ASXL subunits link MBD5 and MBD6 to BAP1 via the C-terminal PHD fingers

**Fig. 2:**
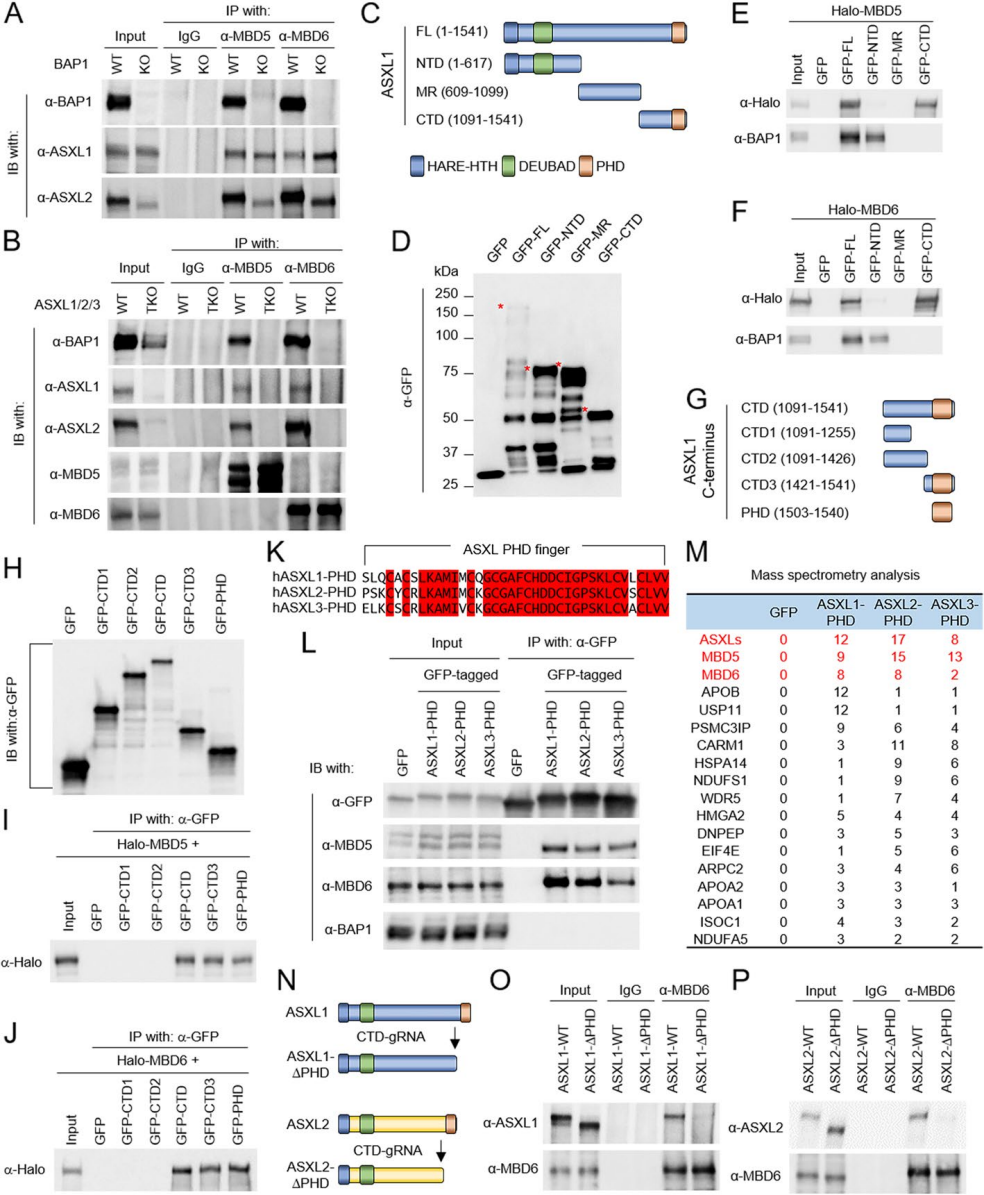
The ASXL subunits link MBD5, MBD6 to BAP1 via their C-terminal PHD fingers. **A** IP of endogenous MBD5 or MBD6 from wild-type or BAP1 knockout HEK293T cells followed by IB for BAP1, ASXL1, and ASXL2. IgG was used as a negative control, *n* = 2. **B** IP of endogenous MBD5 or MBD6 from ASXL1/2/3 triple-knockout (TKO) HEK293T cells followed by IB for BAP1, ASXL1, ASXL2, MBD5, and MBD6. IgG was used as a negative control, *n* = 2. **C** Schematic diagram depicting the truncations of human ASXL1 protein. **D** HEK293T cells were transfected with plasmids expressing GFP, GFP-tagged full-length, or truncated ASXL1. The levels of GFP-tagged proteins were determined by western blot analysis, *n* = 2. **E**, **F** HEK293T cells were co-transfected with plasmids expressing each fragment of ASXL1 and either Halo-tagged MBD5 (**E**) or MBD6 (**F**). Then, the cells were subjected to co-IP assay using GFP-trap agarose followed by western blotting using antibodies against BAP1 and Halo-tag, *n* = 2. **G** Schematic diagram depicting the truncations of C-terminus of human ASXL1 protein. **H** HEK293T cells were transfected with plasmids expressing either GFP, GFP-tagged full-length, or GFP-tagged truncated C-terminus of ASXL1 and the protein levels of GFP-tagged proteins were determined by western blot analysis, *n* = 2. **I**, **J** HEK293T cells were co-transfected with plasmids expressing GFP-tagged truncated C-terminus of ASXL1 and either Halo-tagged MBD6 (**I**) or MBD5 (**J**). They were subjected to co-IP assay using an antibody against GFP followed by western blotting using antibodies against Halo-tag, *n* = 2. **K** CLUSTALW alignment shows the similarity between PHD fingers of ASXL1/2/3. **L** HEK293T cells were transfected with GFP-tagged PHD fingers of ASXL1/2/3 followed by IP of GFP and IB of MBD5, MBD6, and BAP1, *n* = 2. **M** The GFP-fusion proteins were purified from HEK293T cells transduced with GFP-tagged PHD finger of ASXL1/2/3. The purified proteins were subjected to mass spectrometry analysis. Peptide numbers of representative proteins were shown. **N** Schematic diagram depicting the PHD finger depletion of human ASXL1/2 proteins targeted by sgRNA. **O**, **P** IP of MBD6 from wild-type cells and ASXL1-PHD finger deleted (**O**) or ASXL2-PHD finger deleted (**P**) cells followed by IB of MBD6 and ASXL1 (**O**) or ASXL2 (**P**), *n* = 2

To investigate how MBD5 and MBD6 subunits are incorporated into the BAP1 complex, we first hypothesized that MBD5 and MBD6 may directly interact with BAP1 itself, similar to how other subunits function (e.g., ASXL1-3 and FOXK1/2). Therefore, we conducted an immunoprecipitation experiment to determine whether there is an interaction between MBD5, MBD6, and ASXL1/2 subunits in BAP1 knockout HEK293T cells. Surprisingly, both MBD5 and MBD6 can still interact with ASXL1 and ASXL2 in the absence of BAP1 (Fig. 2A). This result indicates an indirect interaction between MBD5, MBD6, and BAP1. Based on these studies published by our own lab and other groups, the ASXL proteins usually function as scaffold proteins that link BAP1 to other epigenetic factors. Therefore, to determine whether ASXLs play a role in mediating the interaction between MBD5, MBD6, and BAP1, we knocked out all three ASXL genes (TKO) by CRISPR in HEK293T cells. Surprisingly, as shown in Fig. 2B, the protein-protein interaction between MBD5, MBD6, and BAP1 is completely abolished in ASXL-TKO cells (Fig. 2B). Due to the high similarity between all three ASXL proteins, we sought to map the interaction domain between MBD5, MBD6, and ASXLs by truncating ASXL1 into N-terminus (NTD), Middle-region (MR), and C-terminus (CTD) fragments (Fig. 2C, D). We then co-transfected HEK293T cells with plasmids expressing the above GFP-tagged fragments of ASXL1 and either Halo-tagged MBD5 or MBD6. As a result, we found that both MBD5 (Fig. 2E) and MBD6 (Fig. 2F) interact with the C-terminus of ASXL1, while BAP1 interacts with ASXL1 N-terminus, which is consistent with previously reported studies [28].

In order to narrow down and identify a more precise protein-binding interface within the ASXL1-CTD that could interact with between MBD5 and MBD6, additional truncations within the C-terminus of ASXL1 were subject to immunoprecipitation experiments (Fig. 2G, H). As a result, we have identified the PHD finger domain at the very C-terminal end of ASXL1 protein could directly interact with both MBD5 (Fig. 2I) and MBD6 (Fig. 2J). In addition, a similar protein-protein interaction was observed between endogenous MBD5, MBD6, and ASXL2 or ASXL3 PHD fingers, since the PHD fingers are highly conserved between ASXL1-3 (Fig. 2K, L). This result was further confirmed by the GFP-tagged ASXLs-PHD finger purification and the mass spectrometry experiment (Fig. 2M, Additional file 3: Table S2).

Finally, to determine whether the PHD fingers of endogenous ASXL proteins link MBD5 and MBD6 to the BAP1 complex in cells, we designed sgRNA targeting the region prior to PHD fingers of ASXL1 and ASXL2 (Fig. 2N). Depletion of the PHD fingers did not affect the stability of ASXL1 or ASXL2, or the interaction between ASXL1, ASXL2, and BAP1 (Additional file 2: Fig. S2A, B). However, the removal of the PHD fingers completely blocked the interaction between endogenous MBD6 and ASXL1/ASXL2 proteins (Fig. 2O, P). Previously, it has been demonstrated that the PHD fingers of ASXL proteins do not contribute to their chromatin binding ability [16, 29]. Therefore, our results have uncovered an important scaffold function of PHD fingers of these ASXL proteins, that is, linking the MBD5 or MBD6 to BAP1, instead of chromatin/histone binding. Interestingly, depletion of ASXL proteins reduced chromatin-bound BAP1 (Additional file 2: Fig. S2C) without affecting the chromatin occupancy of MBD5 or MBD6 (Additional file 2: Fig. S2D), indicating that MBD5 and MBD6 may have an independent chromatin binding ability.

### MBD5 and MBD6 evolutionarily contributes to the stability of the BAP1 complex

**Fig. 3:**
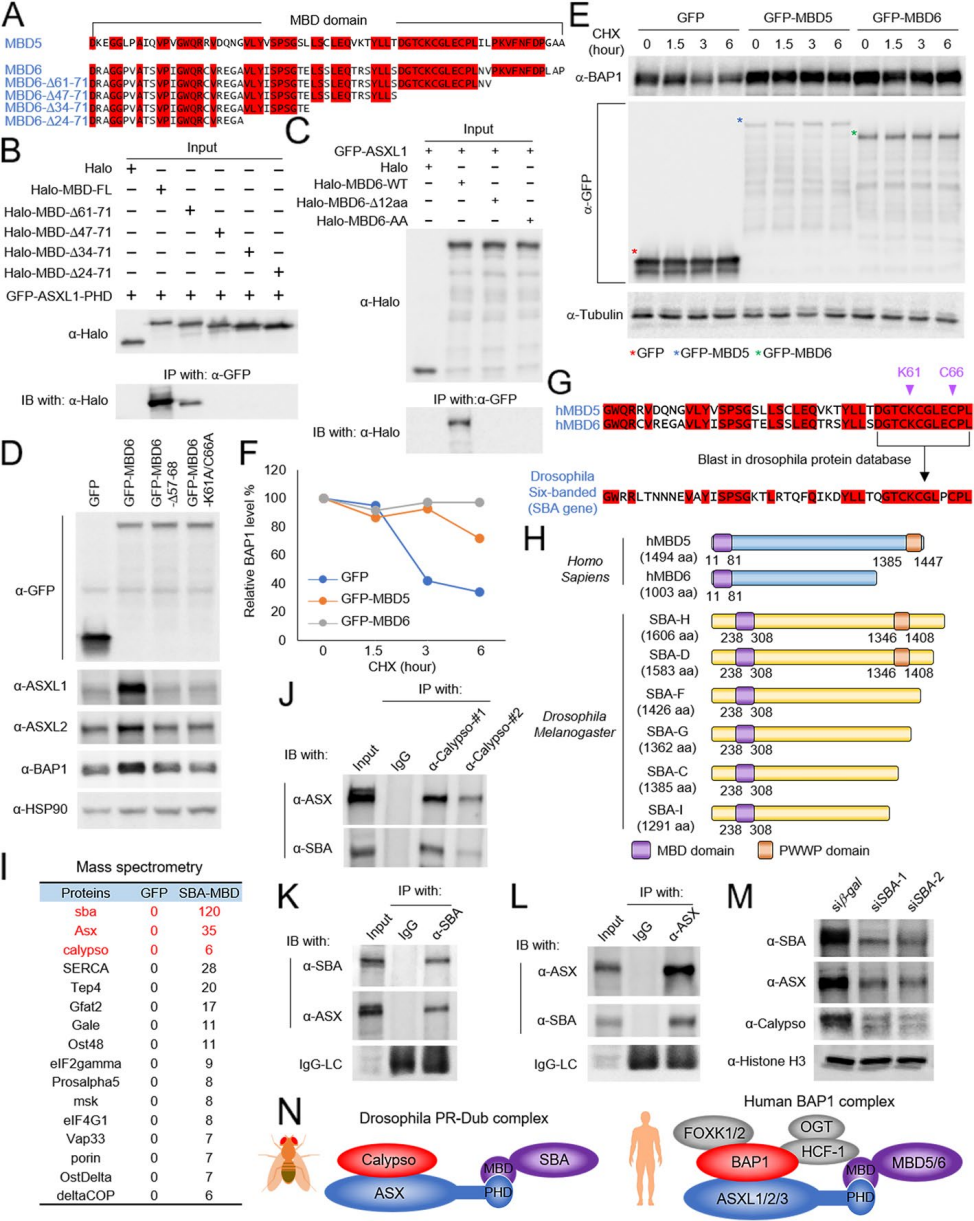
MBD5 and MBD6 evolutionally contributes to the stability of the BAP1 complex. **A** Alignment by CLUSTALW analysis shows the similarity between MBD domains of human MBD5 and MBD6. **B** HEK293T cells were co-transfected with plasmids expressing GFP-tagged PHD finger of ASXL1 and each Halo-tagged truncated MBD domain of MBD6. Then the cells were subjected to co-IP assay using GFP-trap agarose followed by IB for Halo-tag, *n* = 2. **C** HEK293T cells were transfected with plasmids expressing Halo-tagged MBD6-WT, MBD6-Δ12aa, or MBD6-K61A/C66A together with GFP-tagged ASXL1 followed by IP of GFP and IB for Halo-tag, *n* = 2. **D** HEK293T cells were transduced with lentivirus expressing GFP, GFP-tagged MBD6-WT, MBD6-Δ12aa, or MBD6-K61A/C66A. The protein levels of ASXL1, ASXL2, and BAP1 were determined by western blot. HSP90 was used as an internal control, *n* = 2. **E**, **F** HEK293T cells stably expressing GFP, GFP-tagged MBD5, or GFP-tagged MBD6 were treated with CHX (50 μg/ml) for indicated time durations. The whole cell lysate was collected at each time point, and the total protein level of BAP1 was determined by western blot, *n*=2 (**E**) and quantified by ImageJ (**F**). **G** The computational BLAST with the 12-aa from human MBD5 and MBD6 in *Drosophila* protein database. **H** Schematic diagram depicting human MBD5 and MBD6 and isoforms of *Drosophila* SBA. **I** The GFP-fusion proteins were purified from *Drosophila* S2 cells transduced with the GFP-tagged MBD domain of SBA. The purified proteins were subjected to mass spectrometry analysis. Peptide numbers of representative proteins pulled down by the GFP-MBD domain of SBA were shown. **J***Drosophila* S2 cells were subjected to co-IP assay using an antibody against Calypso followed by IB for ASX and SBA, *n* = 2. **K***Drosophila* S2 cells were subjected to co-IP assay using an antibody against SBA followed by IB for SBA and ASX, *n* = 2. **L***Drosophila* S2 cells were subjected to co-IP assay using an antibody against ASX followed by IB for ASX and SBA, *n* = 2. **M** The western blot shows protein levels of SBA, ASX, and Calypso in *Drosophila* S2 cells treated with dsRNA targeting SBA. Histone H3 was used as internal control, *n* = 2. **N** Schema of the *Drosophila* PR-DUB complex and the human BAP1 complex

It has been known that the GFP-tagged MBD5 and MBD6 are not able to sufficiently pull down BAP1 without their N-terminal MBD domain [10]. We confirmed this result by western blot (Additional file 2: Fig. S3A) and further demonstrated that the PHD fingers within ASXL1-3 are sufficient to pull down the MBD domains (Additional file 2: Fig. S3B). Due to high sequence similarities between MBD domains of MBD5 and MBD6 (Fig. 3A), as well as ASXL1-3 PHD fingers (Fig. 2K), we used MBD6 and ASXL1 as an example to identify which amino acid(s) is essential for the protein-protein interaction between MBD proteins and ASXL proteins (Fig. 3A). By immunoprecipitation with a series of MBD domain truncations, we identified a 12-amino acid region, which is 100% conserved between MBD5 and MBD6 and is critical for the interaction with ASXL1 (Fig. 3B).

Based on the protein structure prediction by ColabFold2 [30], which is established on AlphaFold2 and AlphaFold-Multimer (Additional file 2: Fig. S3C), we have identified two conserved amino acids within the MBD domain of MBD6 (K61 and C66), which may be essential for the proper folding ability of the MBD domain and its potential protein-protein interaction. To validate the computational structure predicted, we transfected the HEK293T cells with plasmids expressing Halo-tagged MBD6-WT, MBD6-Δ12aa, or MBD6-K61A/C66A together with GFP-tagged ASXL1 (Fig. 3C). We found that both 12-aa depletion and K61A/C66A double mutations could completely abolish the protein-protein interaction between MBD6 and ASXL1. Indeed, compared to the cells expressing 12aa-depleted or K61A/C66A double mutant MBD6, the cells stably expressing either wild-type MBD5 or MBD6 have higher protein levels of BAP1 and ASXL1/2 (Fig. 3D). Interestingly, in the ASXL-TKO cells, neither MBD5 nor MBD6 could stabilize BAP1 complex (Additional file 2: Fig. S3D). These results revealed a potential role of MBD5 and MBD6 in mediating the stability of the BAP1 complex via their interactions with ASXL1-3. Consistently, we found that both MBD5 and MBD6 can extend the degradation half-life of BAP1 upon CHX treatment (Fig. 3E, F), and significantly reduce the H2AK119Ub levels in a BAP1-dependent manner (Additional file 2: Fig. S3E, F).

The additional sex combs (ASX) protein is conserved from *Drosophila* to mammalian cells [7]. In addition, the *Drosophila* ASX has a very similar PHD finger as human ASXLs at its C-terminus, suggesting that there might also exist a similar MBD domain containing protein in *Drosophila* cells that potentially consists of a relative function resembling human MBD5 and MBD6. To test this hypothesis, we performed the computational BLAST with the 12-aa from human MBD5/MBD6 in the *Drosophila* protein database. Surprisingly, we have identified a *Drosophila* protein, named Six-banded (gene name: *sba*), which contains a highly conserved motif similar to the 12-aa from human MBD5 and MBD6 (Fig. 3G, H). To determine whether the *Drosophila* SBA protein has a similar function to human MBD5 and MBD6 in cells, we purified the MBD domain of SBA from *Drosophila* S2 cells (Additional file 2: Fig. S3G), followed by mass spectrometry analysis (Additional file 4: Table S3). As shown in Fig. 3I, we have successfully detected both ASX and Calypso as binding partners of the SBA-MBD domain. In addition, a strong deubiquitinase activity has been detected by the Ub-AMC assay with the eluted immunoprecipitates (Additional file 2: Fig. S3H). To further determine whether the endogenous SBA/ASX/Calypso forms a complex in *Drosophila* cells, we generated polyclonal antibodies against each of these three proteins and validated the specificity of these antibodies by dsRNA (Additional file 2: Fig. S3I-K). We further performed immunoprecipitation experiments (Fig. 3J–L) and size exclusion experiments (Additional file 2: Fig. S3L) and demonstrated that these three proteins form a complex in *Drosophila* cells. Interestingly, depletion of SBA also reduced the protein levels of ASX and Calypso (Fig. 3M), which is consistent with what we have observed in mammalian cells. In summary, our study has demonstrated that the MBD5 and MBD6 subunits are evolutionarily conserved components within the BAP1 complex and are responsible for maintaining BAP1’s stability (Fig. 3N).

### MBD6 (but not MBD5) is necessary for tumor cell growth

**Fig. 4:**
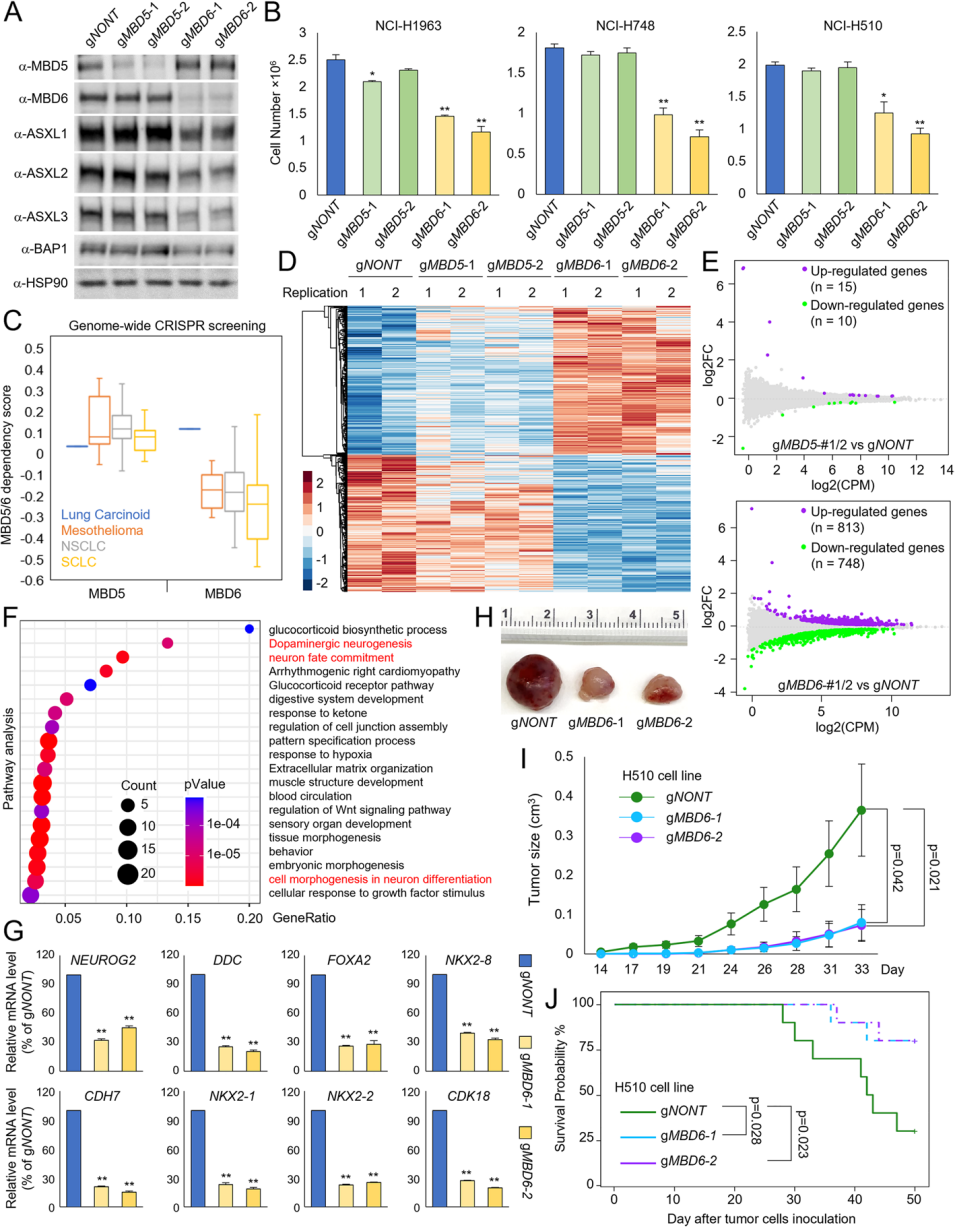
MBD6 but not MBD5 is necessary for tumor cell growth. **A** The human SCLC cell line NCI-H1963 cells were transduced with either non-targeting CRISPR-Cas9 or two distinct CRISPR-Cas9 sgRNAs of MBD5 and MBD6. The protein levels of MBD5, MBD6, ASXL1-3, and BAP1 were determined by western blot, *n*=2. **B** Three different human SCLC cell lines were transduced with either non-targeting CRISPR gRNA, MBD5-specific gRNAs, or MBD6-specific gRNAs for 4 days. The cell numbers were determined by cell counting assay, *n*=3. Two-tailed unpaired Student’s *t* test, **P < 0.01; *P < 0.05. **C** The box plot shows the MBD5 and MBD6 dependency scores of different lung cancer cell types retrieved from the DepMap database. RNA-seq was performed for NCI-H1963 cells transduced with either non-targeting sgRNA, two different MBD5-specific sgRNAs, or two different MBD6-specific sgRNAs. The heatmaps (**D**) and the MA plot (**E**) show the differentially expressed genes by the depletion of MBD5 or MBD6, *n* = 2. **F** Pathway analysis by Metascape of genes that are downregulated upon MBD6 depletion in NCI-H1963 cells. **G** The mRNA levels of *NEUROG2*, *DDC*, *FOXA2*, *NKX2-8*, *CDH7*, *NKX2-1*, *NXK2-2*, and *CDK18* genes, which are all critical genes for SCLC development were determined by real-time qPCR in NCI-H1963 cells transduced with non-targeting sgRNA or two different MBD6-specific sgRNAs, *n*=3. Two-tailed unpaired Student’s *t* test, **P < 0.01; *P < 0.05. **H** 1×10^6^ of NCI-H510 SCLC cells transduced with either non-targeting sgRNAs or two distinct MBD6 sgRNAs that were then inoculated into the right flank of athymic nude mice, *n*=10 per group. Images of representative tumor tissue samples from each mouse were taken at the end of the experiment. **I** The tumor growth was measured using a calibrated caliper every 2–3 days. Weltch’s *t*-test was used for statistical analysis. **J** When each tumor reached 1 cm^3^, the mouse was euthanized and the survival probability was shown. Log-rank test was performed

Emerging studies from other groups as well as our own have revealed a critical oncogenic role of BAP1 in multiple human cancers, including breast cancer [19], leukemia [16, 29, 31], and small cell lung cancer (SCLC) [15, 32]. We have recently shown that depletion of BAP1 by CRISPR or treatment with BAP1 inhibitors could reduce SCLC tumor growth in vitro and in vivo [18]. To study the impact of MBD5 and MBD6 on BAP1’s function in SCLC pathogenesis, we depleted MBD5 or MBD6 with two distinct CRISPR sgRNAs in three different human SCLC cell lines. As shown in Fig. 4A and Fig. S4A, B (Additional file 2: Fig. S4A, B), both of MBD5 and MBD6 have been clearly depleted by western blot. However, we found that there is a significant reduction of all three ASXL proteins as well as BAP1 protein levels in MBD6 but not MBD5-depleted cells. Consistent with this result, depletion of MBD6 (but not MBD5) dramatically reduced cell viability in three different SCLC cell lines (Fig. 4B).

Based on these results, we hypothesized that MBD6 (rather than MBD5) might be the predominant complex form in SCLC cells. To test this, we retrieved the RNA-seq data from 51 human SCLC cell lines and compared the gene expression levels between MBD5 and MBD6. As shown in Fig. S4C and D (Additional file 2: Fig. S4C, D), the average MBD6 mRNA levels are significantly higher than MBD5 in SCLC cells. Then we performed immunoprecipitation experiments in two different SCLC cell lines and determined the endogenous protein-protein interaction between MBD5, MBD6, and other components of the BAP1 complex. As shown in Fig. S4E (Additional file 2: Fig. S4E), we found immunoprecipitation with MBD6-specific antibody is associated with a greater proportion of the BAP1 complex compared to MBD5. These results suggest that MBD6/ASXL/BAP1 composition is more abundant and therefore can be considered as the more predominant form within BAP1 complexes in SCLC cells. Consistently, genome-wide CRISPR screenings by DepMap across different lung cancer types revealed that lung cancer cells are generally more sensitive to MBD6 depletion, while MBD5 is not essential for lung cancer cell viability (Fig. 4C).

To determine how MBD5 and MBD6 can impact transcription in SCLC cells, we conducted RNA-seq in SCLC cells transduced with either non-targeting sgRNA, two distinct MBD5 sgRNAs, or MBD6 sgRNAs. As shown in Fig. 4D and E, depletion of MBD5 leads to mild changes in gene expression within cells, while MBD6 depletion leads to an upregulation of 813 genes and downregulation of 748 genes in both sgRNAs. Pathway analysis has revealed a handful of neuroendocrine genes that are significantly repressed upon MBD6 depletion (Fig. 4F, Additional file 2: Fig. S4F). To further validate the RNA-seq results, we depleted MBD6 with two different CRISPR sgRNAs in three different human SCLC cell lines and performed individual real-time qPCR experiments to determine the mRNA levels of *NEUROG2*, *DDC*, *FOXA2*, *NKX2-8*, *CDH7*, *NKX2-1*, *NXK2-2*, and *CDK18* genes (Fig. 4G, Additional file 2: Fig. S4G, H), which are all critical genes for SCLC development [17, 33, 34]. Finally, to determine the impact of MBD6 on SCLC tumor growth in vivo, we injected NCI-H510 human SCLC cell lines transduced with either non-targeting CRISPR-Cas9 or two distinct MBD6 sgRNAs into the right flank of nude mice. As a result, we found that depletion of MBD6 significantly repressed tumor growth in animals (Fig. 4H, I), and significantly delayed progression of disease (Fig. 4J).

### Characterization of MBD6 occupancy in SCLC cells

In mammalian cells, there are three major BAP1 complexes, which are distinguished by the large scaffold subunits ASXL1, ASXL2, and ASXL3. The ASXL1-3 subunits have mutually exclusive interactions within BAP1 complexes due to sequence similarities. To determine how MBD6 collaborates with these three BAP1 sub-complexes composed of ASXL1-3 in SCLC cell lines, we conducted ChIP-seq experiments to analyze the chromatin occupancy of MBD6 in SCLC cells. Peak annotation revealed that MBD6 is detected at both promoters and enhancers (Additional file 2: Fig. S5A, B). Consistent with our observations seen in HEK293T cells, there is also a significant overlap between MBD6 and BAP1 peaks in NCI-H1963 cells (Additional file 2: Fig. S5C, D). As shown in Fig. S5E, there are 15,057 common peaks between MBD6 and BAP1 via ChIP-seq analysis in NCI-H1963 cells. We further confirmed these results by conducting MBD6 and BAP1 ChIP-seq in a different SCLC cell line NCI-H510 (Additional file 2: Fig. S5F, G) and compared the co-occupancy of MBD6 and BAP1 peaks in the same cell line. As shown in Fig. S5H-J (Additional file 2: Fig. S5H-J), there is also a high co-localization between MBD6 and BAP1 of 10,197 common peaks in NCI-H510 cell lines.

**Fig. 5:**
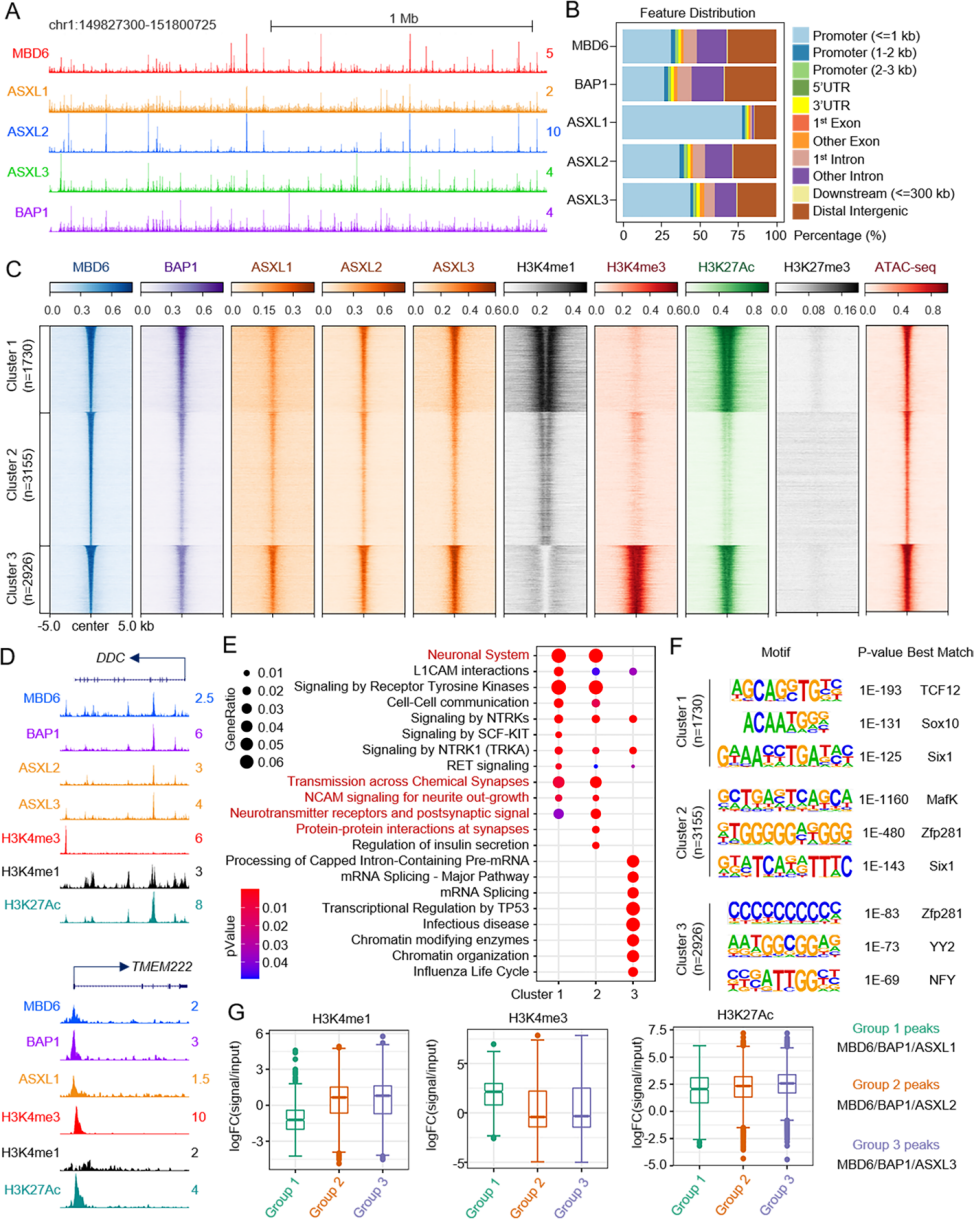
Characterization of MBD6 occupancy in SCLC cells. **A** The representative tracks show chromatin occupancy of MBD6, ASXL1/2/3, and BAP1 binding sites in the human SCLC cell line NCI-H1963. **B** The bar plot shows the feature distribution of MBD6, ASXL1/2/3, and BAP1 peaks in NCI-H1963 cells. **C** Sorted and centered heatmaps generated from ChIP-seq and ATAC-seq data analyses show the occupancy of MBD6, ASXL1/2/3, BAP1, H3K27Ac, H3K4me1, H3K4me3, and H3K27me3 in NCI-H1963 cells. All signals were centered on MBD6 peaks, which were classified into three clusters, based on k-means clustering according to MBD6, H3K4me1, H3K4me3, and H3K27Ac ChIP-seq. **D** Representative tracks showing the occupancy MBD6, BAP1, ASXL1-3, and histone marks. **E** Pathway analysis was performed using ChIPseeker with genes nearest to MBD6 three cluster peaks defined in **C**. **F** Motif enrichment analysis of MBD6 three clusters’ peaks defined in **C** from NCI-H1963 cells. **G** The box plot shows the log2-fold-change of H3K4me1, H3K4me3, and H3K27Ac signals versus input at MBD6/BAP1/ASXL1, MBD6/BAP1/ASXL2, or MBD6/BAP1/ASXL3 co-occupied genomic regions

To further understand the potential genetic interaction between ASXL proteins and MBD6 at the genome-wide level, we conducted ChIP-seq experiments to determine the chromatin occupancy of ASXL1-3 in NCI-H1963 cells. As shown in Fig. 5A and B, we found that both ASXL2 and ASXL3 have similar chromatin binding patterns in comparison to MBD6 in NCI-H1963 cells. Interestingly, the vast majority of ASXL1 peaks are localized at promoters, which is consistent with our previous studies in leukemia and breast cancer cells [8, 16, 35]. In consistency with our ChIP-seq results, the size exclusion experiment has shown significant overlap between ASXL2/3 and MBD6 (Additional file 2: Fig. S5K), indicating that there is a stronger co-function between ASXL2/3 and MBD6 versus ASXL1/MBD6 in SCLC cells. We further conducted ChIP-seq to determine H3K4me1, H3K4me3, H3K27Ac, and H3K27me3 levels in NCI-H1963 cells and centered the peak signals from each of the histone marks into three distinct clusters of MBD6 peaks. As shown in Fig. 5C and D, Cluster 1 and 2 peaks are more enriched with active enhancer markers (e.g., H3K4me1 and H3K27Ac) while Cluster 3 peaks are more enriched with an active promoter marker (H3K4me3). In addition, a significant ATAC-seq signal has been detected at all MBD6 loci (Fig. 5C). These results suggest that MBD6 is associated with histone marks involved with the active promoter and enhancer chromatin regions in SCLC cells. Interestingly, pathway analysis revealed that the genes nearest to Cluster 1 and 2 peaks, which were enriched of TCF12, MafK, and Zfp281 motifs, are involved in neuronal functions (Fig. 5E, F). In contrast, the genes nearest to Cluster 3 peaks are involved in mRNA splicing and metabolism (Fig. 5E, F, Additional file 2: Fig. S5L). Finally, we isolated the common peaks between MBD6/BAP1 and each of the ASXLs (Additional file 2: Fig. S5M) and determined the enrichment of each histone mark present within these three different groups of peaks. As shown in Fig. 5G, the MBD6/BAP1/ASXL1 co-localized peaks are more enriched with H3K4me3 and H3K27Ac, while the MBD6/BAP1/ASXL2 and MBD6/BAP1/ASXL3 co-localized peaks are more enriched with H3K4me1 and H3K27Ac. This indicates that there is a division of labor involving MBD6 on chromatin binding that is dependent on its interaction with distinct ASXLs within BAP1 complexes (Fig. 5B, G).

### MBD6 contributes to BAP1-dependent gene expression in SCLC cells

**Fig. 6:**
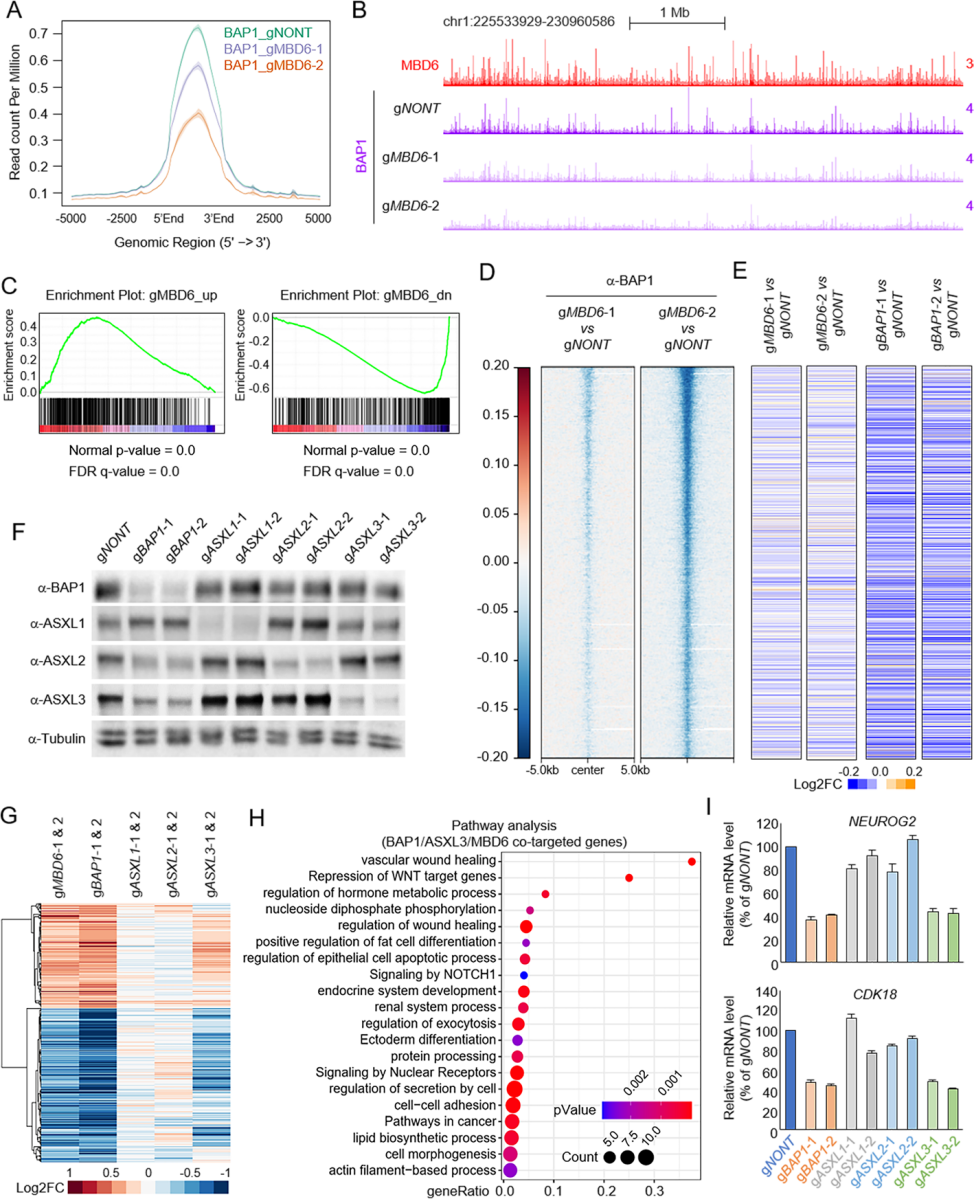
MBD6 is essential for BAP1-dependent gene expression in SCLC. The average plot (**A**) and representative tracks (**B**) show the global reduction of BAP1 in MBD6-depleted SCLC cells by two distinct sgRNAs. **C** Gene Set Enrichment Analysis (GSEA) of BAP1 gene signature enrichment in MBD6-depleted conditions. **D** The log2 fold-change heatmap shows the reduction of BAP1 peaks at MBD6 loci in cells transduced with two distinct MBD6-specific sgRNAs. **E** The log2 fold-change heatmap shows the change of expression levels of genes nearest to MBD6 peaks after MBD6 (left two lanes) and BAP1 (right two lanes) depletion by CRISPR, *n*=2. **F** NCI-H1963 cells were transduced with either non-targeting sgRNA or two distinct sgRNA specific to ASXL1/2/3 or BAP1. The protein levels of BAP1, ASXL1, ASXL2, and ASXL3 were determined by western blot. Tubulin was used as an internal control, *n* = 2. **G** The log2 fold-change gene expression heatmap shows BAP1/MBD6 co-targeted genes in ASXL1/2/3-depleted conditions by CRISPR knockout, *n*=2. **H** Pathway analysis by Metascape of genes that are commonly downregulated upon BAP1, MBD6, or ASXL3 depletion in NCI-H1963 cells. **I** The mRNA levels of *NEUROG2* and *CDK18* were determined by real-time qPCR in NCI-H1963 cells transduced with either non-targeting sgRNA, two different BAP1-specific sgRNAs, or two different ASXL1/2/3 sgRNAs

Based on our previous results (Fig. 4A, Additional file 2: Fig. S4A, B), depletion of MBD6 in SCLC cells leads to a reduction of BAP1 protein levels by western blot. We then sought to determine the impact of MBD6 on BAP1 function involved in transcriptional regulation. As expected, depletion of MBD6 by two distinct sgRNAs significantly reduced BAP1 occupancy at the genome-wide level (Fig. 6A, B). RNA-seq analysis revealed that there is a significant proportion of genes that are co-regulated by MBD6 and BAP1 that are involved in several signaling pathways and cell morphological/developmental characterization (Additional file 2: Fig. S6A, B). To extend this observation on a more precise scale, we employed Gene Set Enrichment Analysis (GSEA) of BAP1 gene signature enrichment in MBD6-depleted conditions (Fig. 6C). The BAP1 signature genes were significantly positively correlated with MBD6 signature genes. Then, we incorporated the RNA-seq data from MBD6 or BAP1-depleted cells with BAP1 ChIP-seq results. As shown in Fig. 6D and E, we found loss of MBD6 dramatically reduced BAP1 occupancy as well as the expression of the genes nearest to BAP1 peaks. To determine which ASXL protein is involved in MBD6/BAP1-mediated gene expression in SCLC cells, we depleted each of the ASXL genes by using two distinct CRISPR sgRNAs per ASXL (Fig. 6F). Interestingly, by RNA-seq analysis, we found that the gene expression profile of ASXL3, but not ASXL1 or ASXL2-depleted cells, is similar to BAP1 and MBD6-depleted cells (Fig. 6G–I, Additional file 2: Fig. S6C). Consistent with the RNA-seq results, genome-wide ChIP-seq analysis revealed that depletion of MBD6 also lead to a global reduction of ASXL3 chromatin occupancy (Additional file 2: Fig. S6D). In addition, restoration of wild-type but not K61A/C66A mutated MBD6 in the MBD6-depleted SCLC cells could at least partially rescue the cell growth ability and gene expression (Additional file 2: Fig. S6E-H). Overall, these results indicate that the BAP1/ASXL3/MBD6 axis is crucial for maintaining SCLC-dependent gene expression and therefore can be considered as a potential epigenetic target for SCLC therapy.

## Discussion

In mammals, the family of MBD proteins consists of eleven proteins that all share the MBD domain [36–38]. MBD5 and MBD6 are the most recently discovered, and thus, their properties remain poorly characterized. For instance, contrary to its name, the MBD domain of MBD5 and MBD6 has been previously shown not to bind to methylated DNA and therefore its function has remained unclear [39]. Previously, a few studies have identified MBD5 and MBD6 as BAP1-associated proteins during in vitro protein purification [10]. However, they all relied on a single study in which the interaction between them was confirmed by IP-MS using cells expressing tagged MBD5, MBD6, or tagged BAP1 complex subunits. In addition, how MBD5 and MBD6 proteins impact the BAP1 complex’s function has not been elucidated. Therefore, MBD5 and MBD6 proteins have not yet been recognized as stable subunits within the BAP1 complex based on the existing literature [12, 22–25].

In our current studies, by generating MBD5 and MBD6-specific antibodies, we have successfully detected the protein-protein interaction between endogenous MBD5, MBD6, and the BAP1 complex. Furthermore, we have demonstrated that MBD5 or MBD6 do not directly interact with BAP1; instead, they bind to the C-terminal PHD fingers of all three ASXL subunits (ASXL1-3). Surprisingly, by protein database BLAST, we have identified an evolutionarily conserved orthologue in *Drosophila*, the six-banded protein (SBA), which also functions as a subunit within the Calypso/ASX complex. It has been shown that the C-terminus of the ASX protein was degraded/truncated during the TAP-Calypso purification in *Drosophila* cells [7]; therefore, this explains why SBA has not yet been identified in previous Calypso purification by other groups.

The *SBA* gene was named based on its embryonic expression pattern in *Drosophila* and was first identified and characterized as a *Drosophila* P-element enhancer detector insertion (F125), which is expressed in the embryonic head and CNS as well as in various third instar imaginal discs [40]. Overexpressing of SBA in *Drosophila* has a strong synergistic effect with euchromatic histone-lysine N-methyltransferase (EHMT), resulting in a consistent disruption of vein patterning and a strong increase in ectopic vein formation [41]. Therefore, we propose to rename *Drosophila SBA* gene as *dMBD5/6*, and how Calypso is involved in *dMBD5/6*-mediated transcriptional regulation warrants further investigation in the future.

The PHD finger is a small protein domain of 50–80 amino acid residues of diverse sequences containing a zinc-binding motif. More than 100 human proteins have this domain and recently it has been reported that some types of PHD finger bind to unmodified or methylated states of histone tail [42]. However, it has been shown that the PHD fingers of ASXLs are not essential for their chromatin-bound ability [16, 29], suggesting that the ASXL-PHD fingers may have a distinct function other than being readers of histone modifications. Indeed, we found that the ASXL-PHD fingers function as linkers that bridge MBD5 and MBD6 to the BAP1 complex. Therefore, disease-specific mutations that truncated the C-terminus of ASXL or induced ASXL N-terminal fusions may disrupt the interaction between MBD5, MBD6, and the BAP1 complex.

Emerging studies have revealed that the dysregulation or mutations within the BAP1 complex were demonstrated to be critical for tumorigenesis. Our recent studies have uncovered an essential oncogenic function of BAP1 in human small cell lung cancer (SCLC) [18]. Depletion of BAP1 or treatment with BAP1-specific inhibitors could significantly reduce SCLC tumor growth in vitro and in vivo [18]. In our current studies, we found that the expression of MBD6 is consistently higher than MBD5 across 51 SCLC cell lines, and functions as the predominant form in the BAP1 complex. Indeed, based on genome-wide CRISPR screening data, we found that MBD6 but not MBD5 is essential for the cell viability of SCLC, as well as BAP1’s stability. This result is further supported by the ChIP-seq and RNA-seq data results showing that MBD6 is also essential for the levels of chromatin-bound BAP1 to promote BAP1-dependent gene expression in SCLC cells.

Mutations within MBD5 and MBD6 have been detected in multiple human cancers, as well as neuronal disorders [43, 44]. Based on these studies from other groups and our current studies, the N-terminal MBD domains within MBD5 and MBD6 are critical for the stability of the BAP1 complex. Therefore, truncated mutations that produce a more stable N-terminus of either MBD5 or MBD6 may lead to a hyper-stabilized/activated BAP1 complex, resulting in aberrant transcriptional reprogramming.

## Conclusions

In this study, we have discovered the function of MBD5 and MBD6 subunits within the BAP1 complex. Mechanistically, MBD5 and MBD6 directly binds to the C-terminal PHD fingers of ASXL1-3, which may function as a potential degron domain that determines the stability of BAP1/ASXL complexes. We further discovered that in context of BAP1-dependent SCLC, MBD6 predominantly functions over MBD5 as being critical for the maintenance of BAP1 function and overall stability at the genome-wide level. Consequently, loss of MBD6 significantly reduces SCLC tumor growth in vitro and in vivo, and therefore, our study may provide potential targets for SCLC clinical therapies.

## Methods

### Antibodies and reagents

BAP1 (#13271S), ASXL2 (#71257), H3K27ac (#8173S), H3K4me1 (#5326S), H3K4me3 (#9751), H3K27me3 (#9733), and histone H3 (#4499S) antibodies were purchased from Cell Signaling Technology. HSP90 (sc-7947) and GFP (sc-9996) antibodies were purchased from Santa Cruz. MBD5 (ABE1322) antibody (For western blot) was purchased from Millipore. Tubulin antibody (E7) was purchased from Developmental Studies Hybridoma Bank. Halo-tag (G9211) antibody was purchased from Promega. Anti-ASXL1, ASXL3, and BAP1 (for ChIP-seq) were previously described. Anti-ASXL2 (For ChIP-seq) was generated against human ASXL2 amino acids 387-546. Anti-MBD5 (For ChIP-seq) was generated against human MBD5 amino acids 230-520. Anti-MBD6 (For western blot, IP, and ChIP-seq) was generated against human MBD6 amino acids 144-299. Anti-Calypso (For western blot and IP) was generated against full-length *Drosophila* Calypso. Anti-ASX (For western blot and IP) was generated against *Drosophila* ASX amino acids 404-639. Anti-SBA (For western blot and IP) was generated against *Drosophila* SBA amino acids 234-479.

### Plasmids

The Halo-tagged plasmids pFN21A-MBD5 (FHC05942) and pFN21A-MBD6 (FHC11608) were purchased from Promega. The ASXL1-NTD, ASXL1-MR, ASXL1-CTD, ASXL1-CTD1, ASXL1-CTD2, ASXL1-CTD3, ASXL1-PHD, ASXL2-PHD, and ASXL3-PHD DNA were either subcloned from full-length ASXL1 or synthesized as gBlock fragment DNA (Integrated DNA Technologies, Inc.), and inserted into pLJM1-GFP vector. The DNA of MBD5-MBD domain, MBD6-MBD domain, MBD6-MBD-Δ61-71, MBD6-MBD-Δ47-71, MBD6-MBD-Δ34-71, and MBD6-MBD-Δ24-71 were subcloned from either pFN21A-MBD5 or pFN21A-MBD6 vector and inserted into pLJM1-Halo vector. The gBlock fragment DNA of SBA-MBD domain was synthesized at IDT and inserted into pAc5.1B-EGFP vector. The primers and gBlock sequence for the subcloning are listed in Supplementary Table 4 (Additional file 5: Table S4).

### Cell lines

HEK293T cells were obtained from ATCC, and then maintained with DMEM (Gibco, Gaithersburg, MD) containing 10% FBS (Sigma). The SCLC cell lines were obtained from ATCC. NCI-H748, NCI-H1963, and NCI-H510 cells were maintained with ATCC-formulated RPMI-1640 medium containing 10% FBS (Sigma). The *Drosophila* S2 cells were maintained in HyClone™ SFX-Insect Cell Culture Media containing 10% FBS (Sigma).

### Mouse experiments

All mouse work was performed in accordance with protocols approved by The Center for Comparative Medicine (CCM) of Northwestern University. Five- to six-week-old athymic nude mice were used for xenograft experiments. For tumor growth assays, 1 × 10^6^ human SCLC cell line NCI-H510 cells were transduced with either non-targeting CRISPR sgRNA or two distinct MBD6-specific CRISPR sgRNAs and then inoculated into the right flank of nude mice. Tumor growth was monitored every 2 to 3 days for 2 weeks after inoculation.

### Immunoprecipitation (IP)

The IP experiment was performed as described before [15]. Briefly, the cells were lysed in the lysis buffer (50mM Tris pH 8.0, 150 mM NaCl, 0.5% Triton X100, 10% Glycerol, protease inhibitors, and benzonase). After centrifugation at max speed at 4°C for 15 min, the supernatants were collected and incubated with the primary antibody and Protein A/G (Santa Cruz) at 4°C overnight with rotation. Then the samples were washed with lysis buffer four times and boiled in 6 × SDS sample loading buffer.

### RNA interference, CRISPR-mediated knockouts, and real-time PCR

Designed sgRNAs were cloned into lentiCRISPR v2 (Addgene, 52961) vector. Lentiviral-mediated CRISPR/Cas9 knockout was described previously [18]. For RNA interference in *Drosophila* cells, the S2 cells are maintained at 2×10^6^ cells/ml in serum-free SFX medium (containing 1% penicillin/streptomycin) prior to RNAi treatment. Then, the cells were plated at 5×10^5^ cells/ml in 20 ml SFX per T75 flask, followed by treatment with 100 μg dsRNA for 5–6 days. Oligo sequences used in this manuscript are listed in Supplementary Table 4 (Additional file 5: Table S4).

### RNA-seq and analysis

RNA-seq was conducted as previously described [15]. Paramagnetic beads coupled with oligo d(T) are combined with total RNA to isolate poly(A)+ transcripts based on NEBNext® Poly(A) mRNA Magnetic Isolation Module manual. All remaining steps for library construction were used according to the manufacturer’s recommendations. Samples were pooled and sequenced on a HiSeq with a read length configuration of 150 PE. Gene counts were computed by HTSeq and used as an input for edgeR 3.0.85257. Genes with Benjamini-Hochburg adjusted *p*-values less than 0.01 were considered to be differentially expressed (unless otherwise specified).

### ChIP-seq assay and analysis

ChIP-seq was performed as described previously [15]. For histone modifications, 10% of Drosophila chromatin was used as spike-in control. For ChIP-seq analysis, all the peaks were called with the MACS v2.1.0 software using default parameters and corresponding input samples. Metaplots and heatmaps were generated using ngsplot database to display ChIP-seq signals. Peak annotation, motif analysis, and super enhancer analyses were performed with HOMER and ChIPseeker. Pathway analysis was performed with Metascape and ChIPseeker.

### Mass spectrometry sample preparation and analysis

Mass spectrometry was performed as described previously [15]. Protein pellet was denatured in 50 μL of 8 M Urea/0.4 M Ammonium Bicarbonate followed by reduction in 2 μL of 100 mM DTT. The digests were acidified to 0.5% trifluoroacetic acid (TFA), and the peptides were then desalted on C18 Sep-Paks (Waters). The pooled extracts were dried in a vacuum concentrator and resuspended in 30 uL of 5% ACN/0.1% FA for LC-MS analysis. Peptides were analyzed by LC-MS/MS using a Dionex UltiMate 3000 Rapid Separation LC (RSLC) system and a linear ion trap-Orbitrap hybrid Elite mass spectrometer (Thermo Fisher Scientific Inc, San Jose, CA).

### Statistical analyses

For statistical analyses, GraphPad Prism 7, Microsoft Excel, and R were used [15]. All data involving a statistical analysis being reported met the criteria to use the appropriate statistical tests. For the normal distribution of data, the empirical rule was used to infer the distribution. For growth curves and time-course, RNA-seq *t*-tests were calculated between the area-under-the-curve (AUC) values. Statistical tests used are reported in the figure legends.

## Supplementary Information

Additional file 1: Table S1. Mass spectrometry results for GFP-tagged MBD5 and MBD6 purification from HEK293T cells.

Additional file 2: Fig S1. Generation of polyclonal antibodies against MBD5 and MBD6. Fig S2. The ASXL subunits link MBD5 and MBD6 to BAP1 complex via the C-terminal PHD fingers. Fig S3. MBD5 and MBD6 evolutionally contributes to stability of BAP1 complex. Fig S4. MBD6 but not MBD5 is critical for SCLC cell viability. Fig S5. Characterization of MBD6 occupancy in SCLC cells. Fig S6. MBD6 is essential for BAP1-dependent gene expression in SCLC. Fig S7. Uncropped western blot gel images in Fig. 1A and C-E. The dotted line boxes highlight lanes used in figures. Fig S8. Uncropped western blot gel images in Fig. 2A-B, D-F, and H-J. The dotted line boxes highlight lanes used in figures. Fig S9. Uncropped western blot gel images in Fig. 2L-P. The dotted line boxes highlight lanes used in figures. Fig S10. Uncropped western blot gel images in Fig. 3B-E. The dotted line boxes highlight lanes used in figures. Fig S11. Uncropped western blot gel images in Figs. 3J-M and 4A. The dotted line boxes highlight lanes used in figures. Fig S12. Uncropped western blot gel images in Fig. 6F, S1D-E, and S2A-B. The dotted line boxes highlight lanes used in figures. Fig S13. Uncropped western blot gel images in Figure S3A-B, and S3D. The dotted line boxes highlight lanes used in figures. Fig S14. Uncropped western blot gel images in Figure S3E and S3I-L. The dotted line boxes highlight lanes used in figures. Fig S15. Uncropped western blot gel images in Figure S4A and B. The dotted line boxes highlight lanes used in figures. Fig S16. Uncropped western blot gel images in Figure S4E. The dotted line boxes highlight lanes used in figures. Fig S17. Uncropped western blot gel images in Figure S5K and S6F. The dotted line boxes highlight lanes used in figures.

Additional file 3: Table S2. Mass spectrometry results for GFP-tagged PHD fingers of ASXL1-3 purification from HEK293T cells.

Additional file 4: Table S3. Mass spectrometry results for GFP-tagged SBA-MBD purification from drosophila S2 cells.

Additional file 5: Table S4. Primes used for real-time PCR and constructs.

Additional file 6. Review history.

## Data Availability

The RNA-seq data from 51 human SCLC cell lines with refined tumor type annotations were obtained from Cancer Cell Line Encyclopedia Data Portal [45]. The source code of Ceto pipeline used for analyzing the NGS data from this study is available at the Github site: https://github.com/ebartom/NGSbartom [46]. NGS data generated for this study are available at the Gene Expression Omnibus (GEO) under accession number GSE196860 [47]. Mass spectrometry data in this study is included in this manuscript.
