# Supplementary material for: MBD5 and MBD6 stabilize the BAP1 complex and promote BAP1-dependent cancer
Source: Genome Biol. 2022 Sep 30;23:206. doi: 10.1186/s13059-022-02776-x (PMC9523997; doi:10.1186/s13059-022-02776-x)
Supplement: Supplementary file 2 — Additional file 2: Fig S1. Generation of polyclonal antibodies against MBD5 and MBD6. Fig S2. The ASXL subunits link MBD5 and MBD6 to BAP1 complex via the C-terminal PHD fingers. Fig S3. MBD5 and MBD6 evolutionally contributes to stability of BAP1 complex. Fig S4. MBD6 but not MBD5 is critical for SCLC cell viability. Fig S5. Characterization of MBD6 occupancy in SCLC cells. Fig S6. MBD6 is essential for BAP1-dependent gene expression in SCLC. Fig S7. Uncropped western blot gel images in Fig. 1A and C-E. The dotted line boxes highlight lanes used in figures. Fig S8. Uncropped western blot gel images in Fig. 2A-B, D-F, and H-J. The dotted line boxes highlight lanes used in figures. Fig S9. Uncropped western blot gel images in Fig. 2L-P. The dotted line boxes highlight lanes used in figures. Fig S10. Uncropped western blot gel images in Fig. 3B-E. The dotted line boxes highlight lanes used in figures. Fig S11. Uncropped western blot gel images in Figs. 3J-M and 4A. The dotted line boxes highlight lanes used in figures. Fig S12. Uncropped western blot gel images in Fig. 6F, S1D-E, and S2A-B. The dotted line boxes highlight lanes used in figures. Fig S13. Uncropped western blot gel images in Figure S3A-B, and S3D. The dotted line boxes highlight lanes used in figures. Fig S14. Uncropped western blot gel images in Figure S3E and S3I-L. The dotted line boxes highlight lanes used in figures. Fig S15. Uncropped western blot gel images in Figure S4A and B. The dotted line boxes highlight lanes used in figures. Fig S16. Uncropped western blot gel images in Figure S4E. The dotted line boxes highlight lanes used in figures. Fig S17. Uncropped western blot gel images in Figure S5K and S6F. The dotted line boxes highlight lanes used in figures. [file 13059_2022_2776_MOESM2_ESM.docx]

**Supplementary Figure**

**
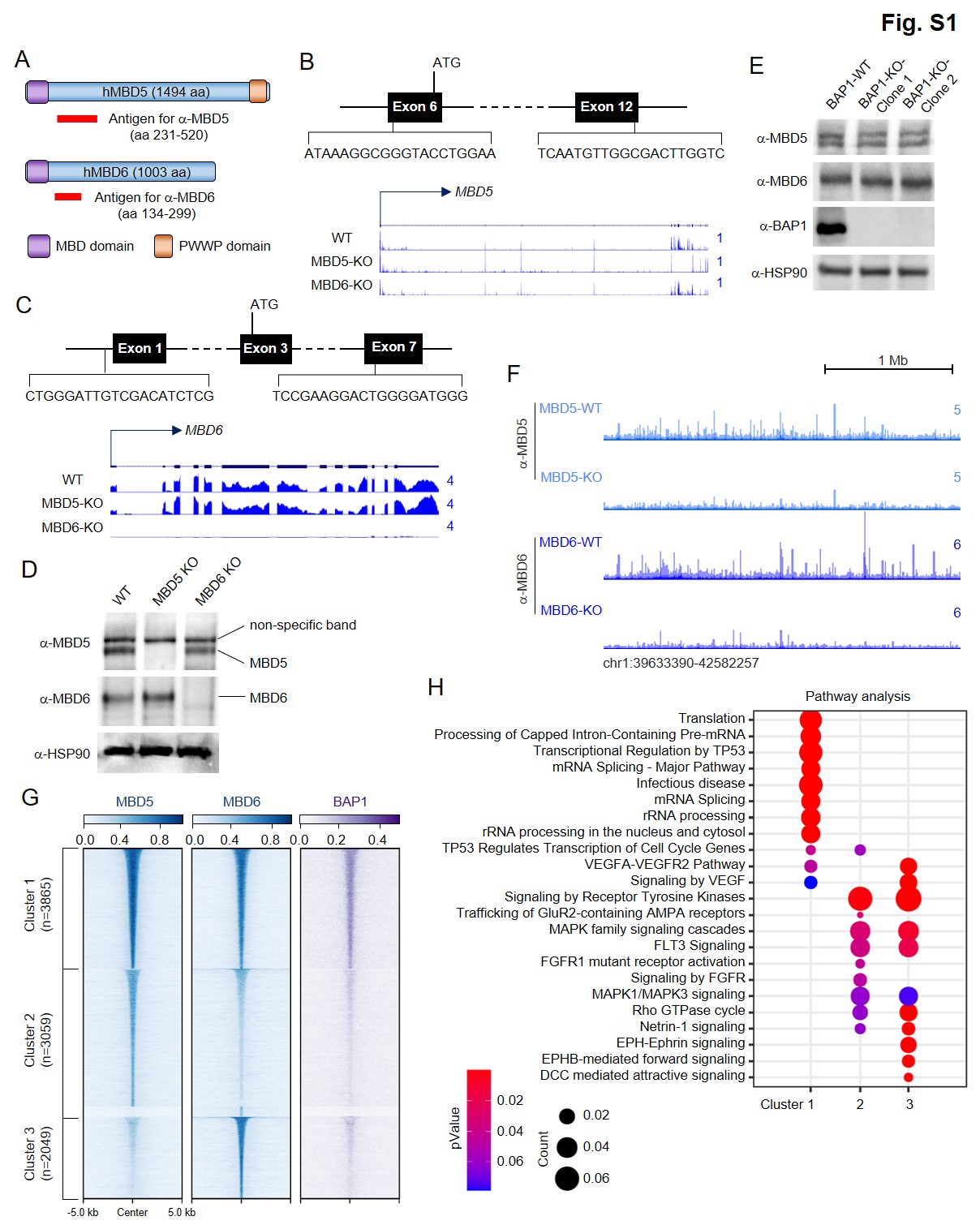
**

**Figure S1. Generation of polyclonal antibodies against MBD5 and MBD6**

A) Schema of human MBD5 and MBD6 protein and the antigen peptide for generation of the polyclonal antibodies. The design of the CRISPR-Cas9 knockout system for MBD5 (B) and MBD6 (C). The gene depletion was validated by RNA-seq. D) The protein levels of MBD5 and MBD6 were determined by western blot in parental cells, MBD5-KO, and MBD6-KO cells. E) The western blot analysis shows the protein levels of MBD5 and MBD6 in BAP1 knockout HEK293T cells. F) The representative tracks show the specificity of home-made MBD5 and MBD6 antibodies, using MBD5 and MBD6-KO cells as negative control. G, H) MBD5 and MBD6 peaks were divided into three clusters based on K-means clustering. The BAP1 peaks were further centered on the three-cluster loci (G). Pathway analysis was performed using ChIPseeker with genes nearest to each group of peaks (H).

**
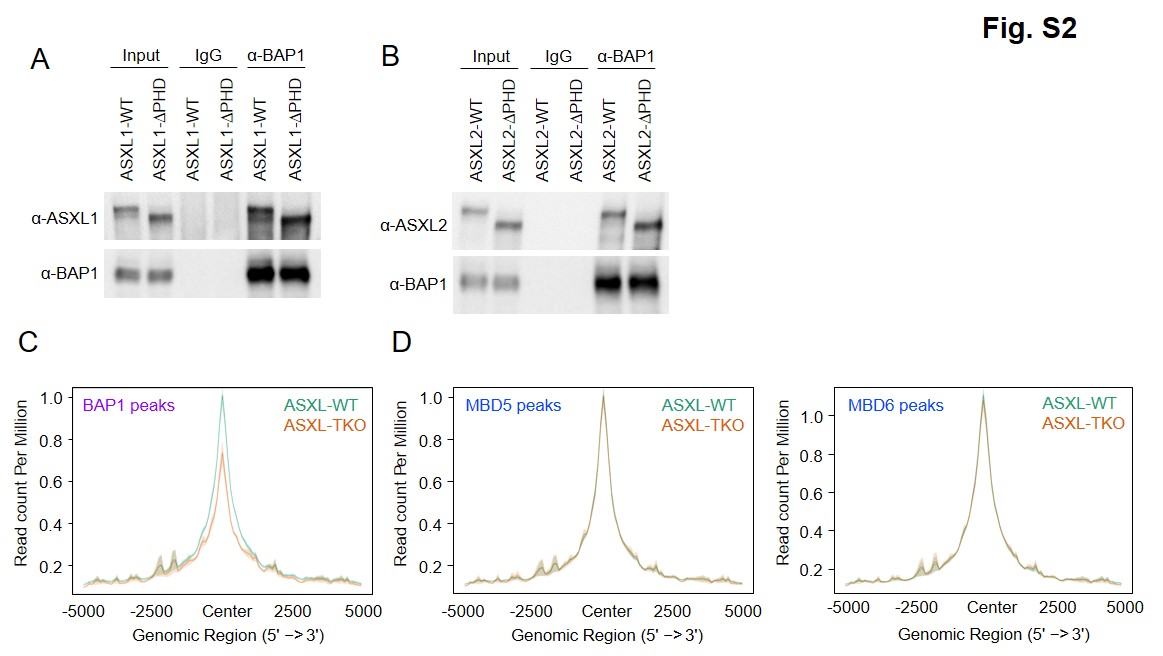
Figure S2. The ASXL subunits link MBD5 and MBD6 to the BAP1 complex via their C-terminal PHD fingers**

A) IP of BAP1 from ASXL1-WT and ASXL1-ΔPHD cells followed by IB of ASXL1 and BAP1, n=2. B) IP of BAP1 from ASXL2-WT and ASXL2-ΔPHD cells followed by IB of ASXL2 and BAP1, n=2. The average plot show the global occupancy of BAP1 (C), MBD5, and MBD6 (D) in ASXL-WT and ASXL-depleted (TKO) HEK293T cells.


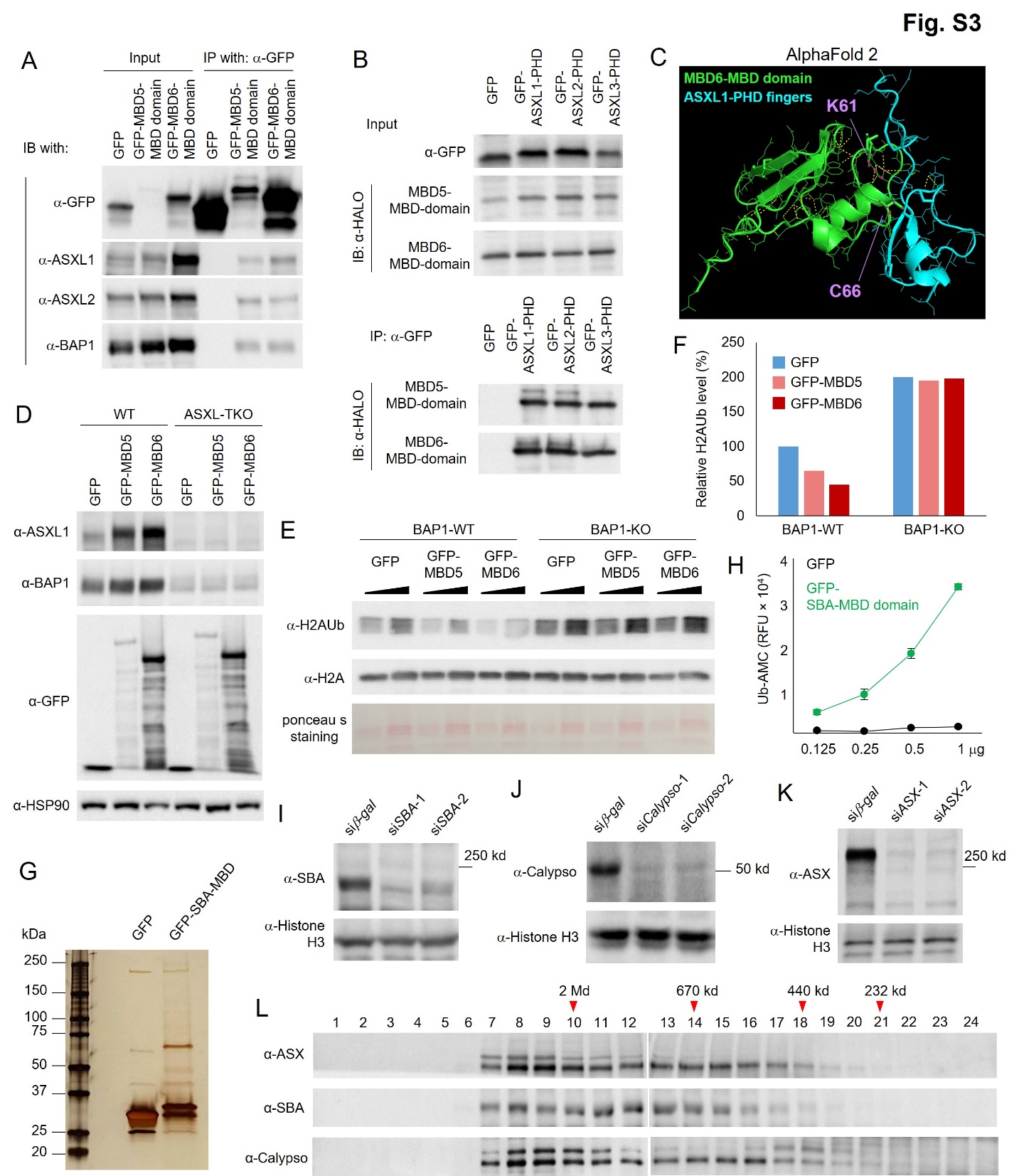


**Figure S3. MBD5 and MBD6 evolutionally contributes to the stability of the BAP1 complex**

A) Immunoprecipitation (IP) was performed with antibodies against the GFP-tag, followed by immunoblotting (IB) with antibodies against ASXL1, ASXL2, and BAP1 in HEK293T cells transfected with plasmids expressing either GFP or GFP-MBD domain of MBD5 and MBD6. IgG was used as a negative control, n = 2. B) HEK293T cells transfected with plasmids expressing Halo-MBD domain of MBD5, MBD6, and either GFP or GFP-tagged PHD finger of ASXL1/2/3 were subjected to IP with GFP trap agarose, followed by IB with antibodies against Halo-tag, n = 2. C) The model represents the interaction between the MBD domain of MBD6 and the PHD finger of ASXL1 created by ColabFold. D) The western blot shows protein levels of ASXL1 and BAP1 in HEK293T-WT and ASXL-TKO cells infected by lentivirus expressing GFP-tagged MBD5 or MBD6. HSP90 was used as an internal control, n = 2. E) The western blot shows protein levels of H2AK119Ub in WT or BAP1-KO HEK293T cells transduced with lentivirus expressing either GFP, GFP-tagged MBD5, or GFP-tagged MBD6 with a gradual increase in the amount of protein loaded per sample. Total H2A and ponceau S staining were used as an internal control, n = 2. F) These results were further quantified by ImageJ. G) Purified GFP-tagged MBD domain of SBA was confirmed by silver staining. H) IP from whole cell lysates were performed with antibody against GFP in *Drosophila* S2 cells transfected with plasmids expressing either GFP or GFP-tagged MBD domain of SBA. The eluted immunoprecipitates were used for the Ub-AMC assay. Homemade polyclonal antibodies against SBA (I), Calypso (J), or ASX (K) were validated by western blot analysis using *Drosophila* S2 cells depleted of each protein by two distinct dsRNAs. L) Nuclear extract from *Drosophila* S2 cells was subjected to SE chromatography and then protein levels of ASX, SBA, and Calypso were determined by western blot analysis, n = 2.

**
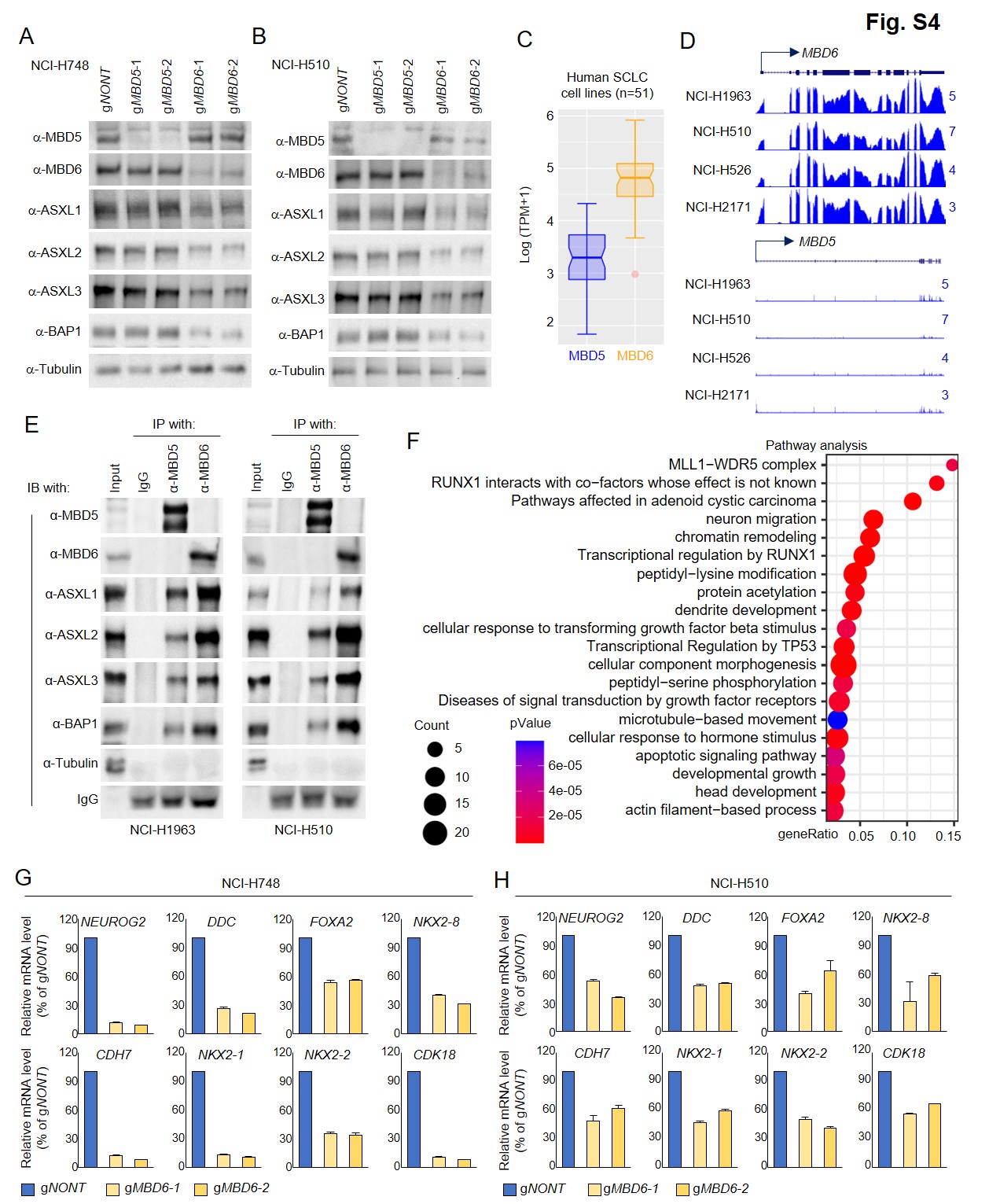
**

**Figure S4. MBD6 (but not MBD5) is critical for SCLC cell viability**

The human SCLC cell line NCI-H748 (A) and NCI-H510 (B) cells were transduced with either non-targeting CRISPR-Cas9 or two distinct CRISPR-Cas9 sgRNAs of MBD5 or MBD6. The protein levels of MBD5, MBD6, ASXL1-3, and BAP1 were determined by western blot, n=2. C) The RNA-seq data from 51 human SCLC cell lines was retrieved from DepMap and the expression levels of MBD5 and MBD6 are shown in the box plot. D) The representative tracks show the mRNA levels of MBD5 and MBD6 in four different SCLC cell lines. E) IP was performed with antibodies against MBD5 or MBD6, followed by IB with antibodies against MBD5, MBD6, ASXL1/2/3, and BAP1 in both NCI-H1963 cells and NCI-H510 cells. IgG was used as a negative control, n = 2. F) Pathway analysis by Metascape of genes that are up-regulated upon MBD6 depletion in NCI-H1963 cells. G, H) The mRNA levels of *NEUROG2*, *DDC*, *FOXA2*, *NKX2-8*, *CDH7*, *NKX2-1*, *NXK2-2*, and *CDK18* genes, which are all critical genes for SCLC development were determined by real-time qPCR in NCI-H748 cells (G) or NCI-H510 cells (H) transduced with either non-targeting sgRNA or two different MBD6-specific sgRNAs, n=3. Two-tailed unpaired Student’s t-test, **P < 0.01; *P < 0.05.


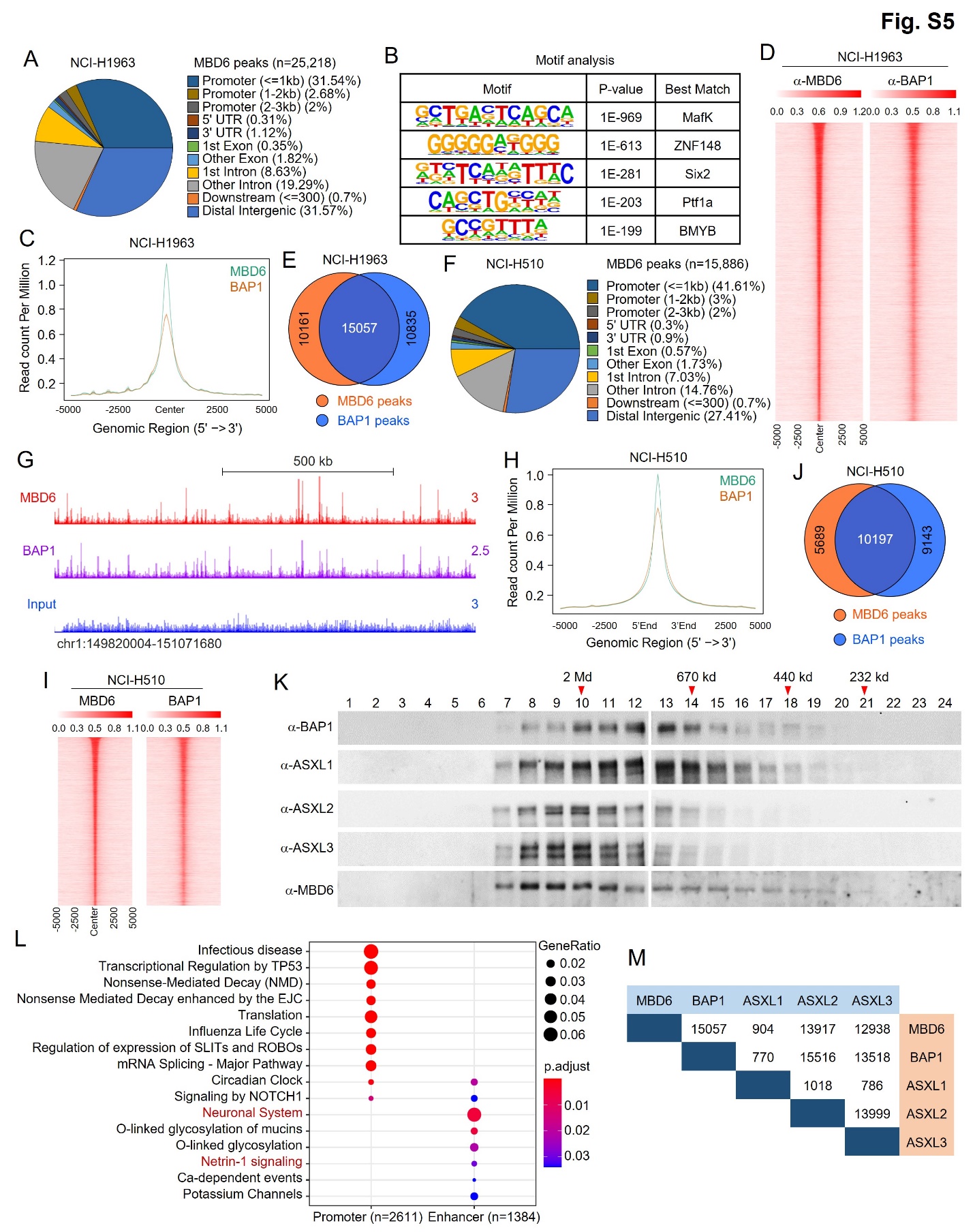


**Figure S5. Characterization of MBD6 occupancy in SCLC cells**

A) Distribution of MBD6 binding to genomic regions in the human SCLC cell line NCI-H1963 as assessed by ChIP-seq. Peak annotation of MBD6 is summarized in a pie chart format. B) Motif enrichment analysis of MBD6 peaks in NCI-H1963 cells. The average plots (C) and occupancy heatmap (D) shows the co-localization of BAP1 and MBD6 peaks in NCI-H1963 cells. Signals were centered on MBD6 peaks. E) The Venn-diagram shows the overlap between MBD6 and BAP1 peaks in NCI-H1963 cells. F) Distribution of MBD6 binding to genomic regions in the human SCLC cell line NCI-H510 as assessed by ChIP-seq. Peak annotation of MBD6 is summarized in the pie chart. The representative tracks (G), average plot (H), and occupancy heat map (I) show the co-localization of BAP1 and MBD6 peaks in NCI-H510 cells. Signals were centered on MBD6 peaks. J) The Venn-diagram shows the overlap of MBD6 peaks and BAP1 peaks in NCI-H510 cells. K) Nuclear extract from NCI-H1963 cells was subjected to SE chromatography and then protein levels of BAP1, MBD6, and ASXL1/2/3 were determined by western blot analysis. n = 2. L) Pathway analysis was performed using ChIPseeker with genes nearest to MBD6-occupied promoter and enhancer regions in NCI-H510 cell lines. M) The table shows the ChIP-seq peaks shared among MBD6, BAP1, and ASXL1/2/3.

**
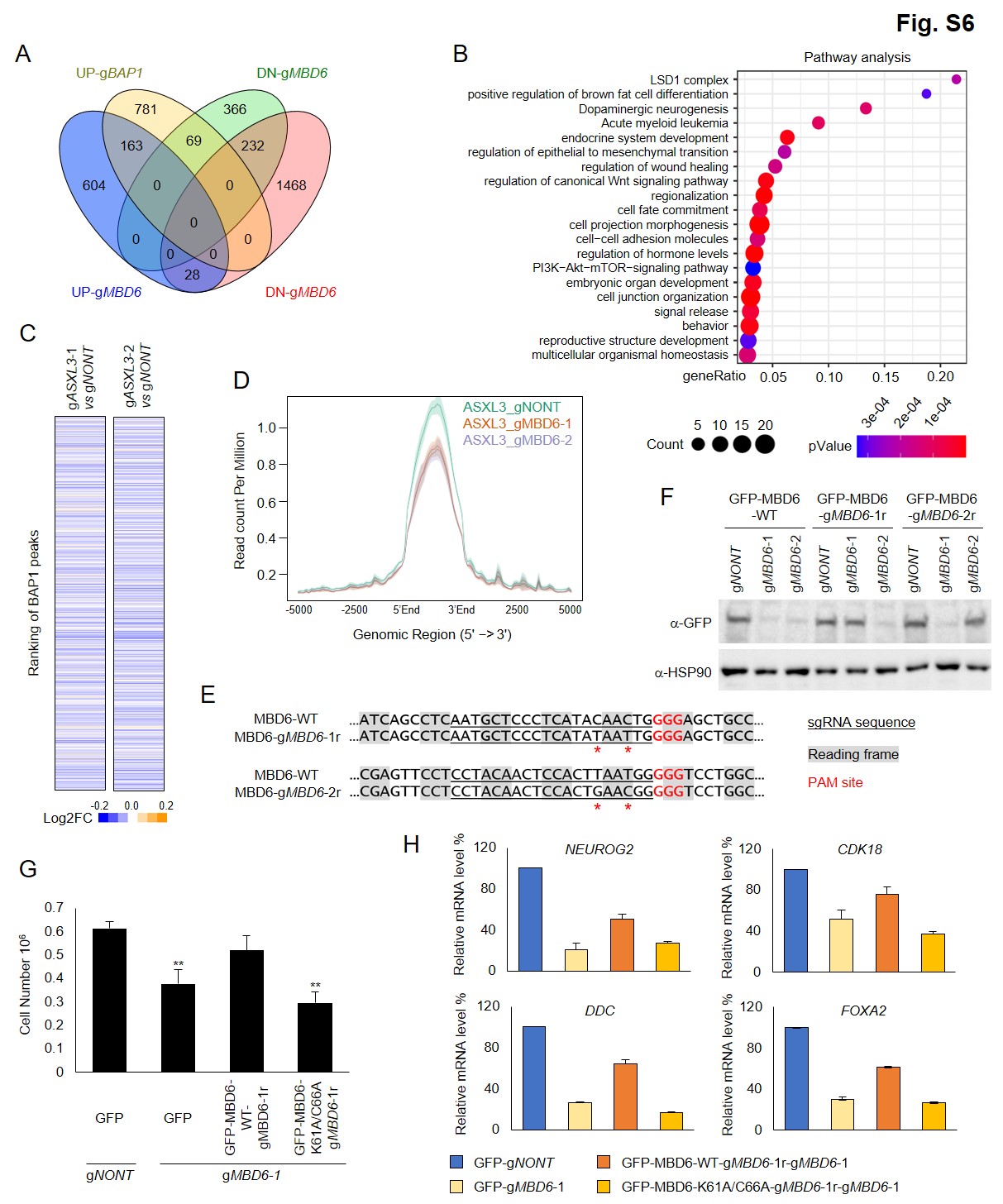
**

**Figure S6. MBD6 is essential for BAP1-dependent gene expression in SCLC**

A) The Venn-diagram analysis shows the overlap of target genes shared between MBD6 and BAP1 in NCI-H1963 cells based on the RNA-seq data. B) Pathway analysis by Metascape of genes that are downregulated in both MBD6 and BAP1-depleted cells (B). C) The log2 fold-change of the expression levels of genes nearest to MBD6 peaks in ASXL3-depleted cells, n=2. D) The average plot shows the global reduction of ASXL3 in MBD6-depleted SCLC cells by two distinct sgRNAs. E) The design of plasmids expressing GFP-tagged CRISPR-resistant (gMBD6r) MBD6. F) The GFP-tagged CRISPR-resistant MBD6 is resistant to CRISPR treatment, n=2. G) GFP, GFP-tagged wild type, or K61A/C66A mutated MBD6 were re-expressed in MBD6 depleted NCI-H510 cell lines, respectively. The cell viability was determined by cell counting assay, n=3. Two-tailed unpaired Student’s t-test, **P < 0.01; *P < 0.05. H) The mRNA levels of MBD6 targeted genes *NEUROG2*, *CDK18*, *DDC*, and *FOXA2* were determined by real-time PCR, n=3.

**
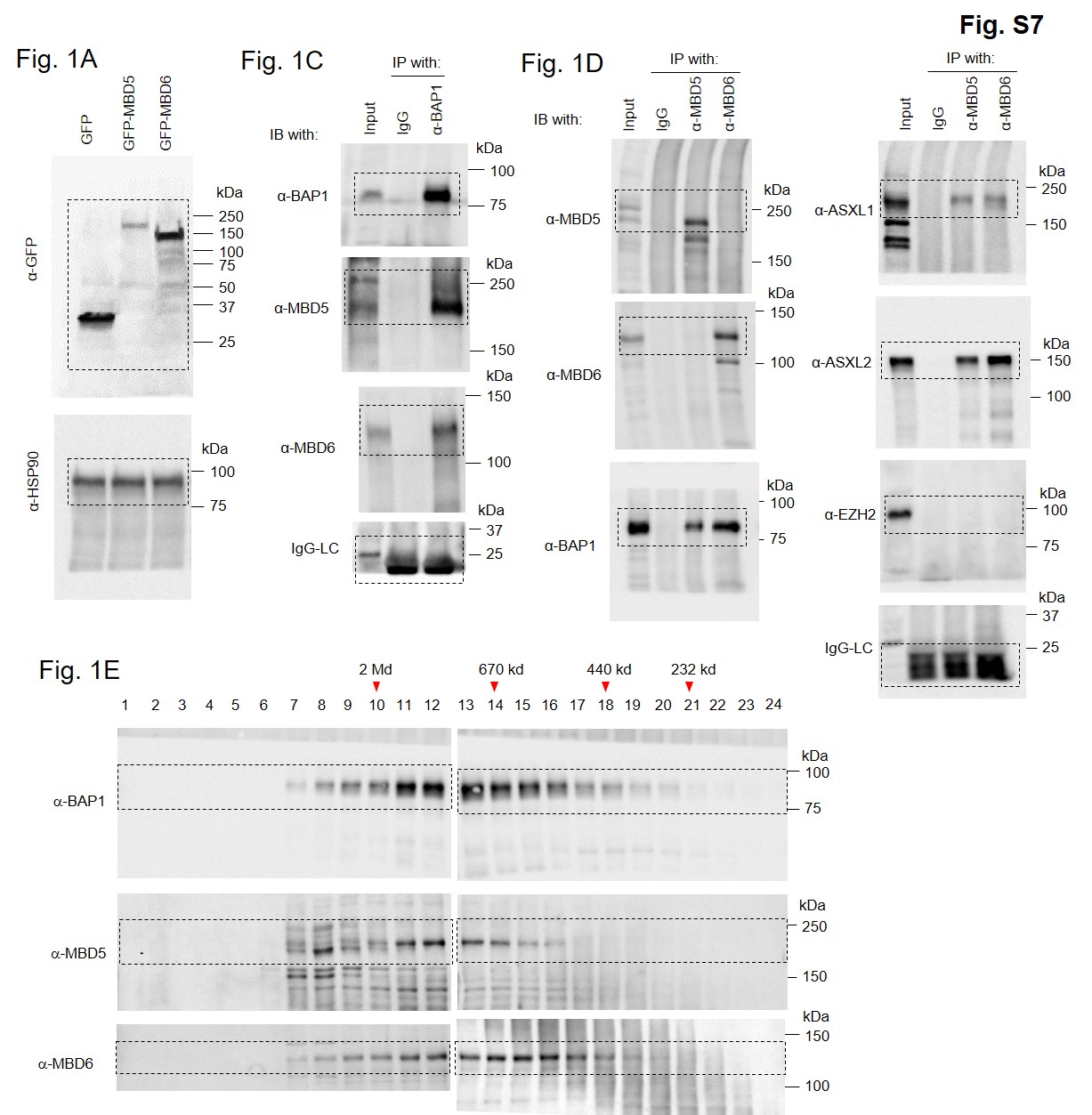
**

**Figure S7. Uncropped western blot gel images in Figure 1A and 1C-E.** The dotted line boxes highlight lanes used in figures.

**
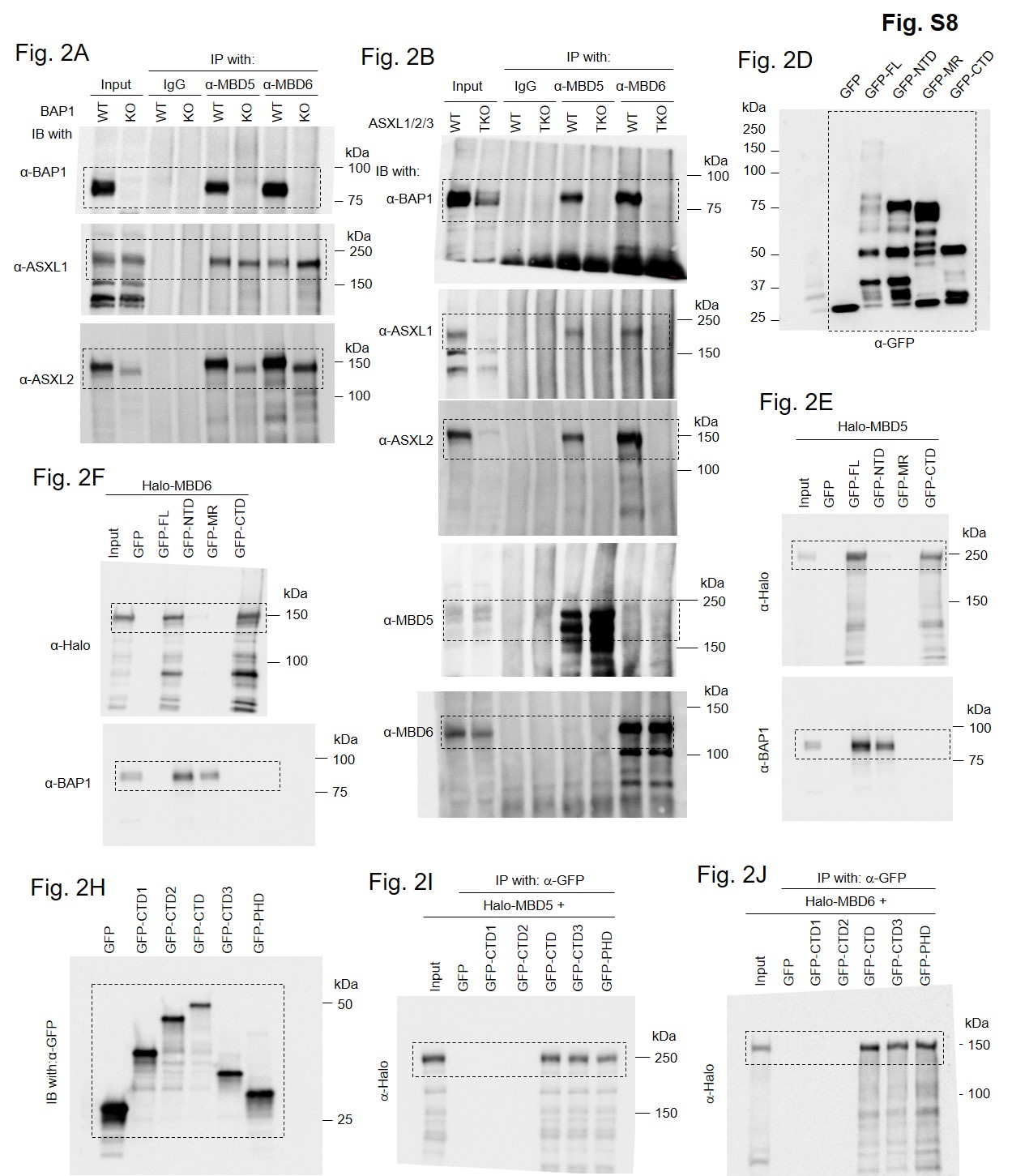
**

**Figure S8. Uncropped western blot gel images in Figure 2A-B, 2D-F, and 2H-J.** The dotted line boxes highlight lanes used in figures.

**
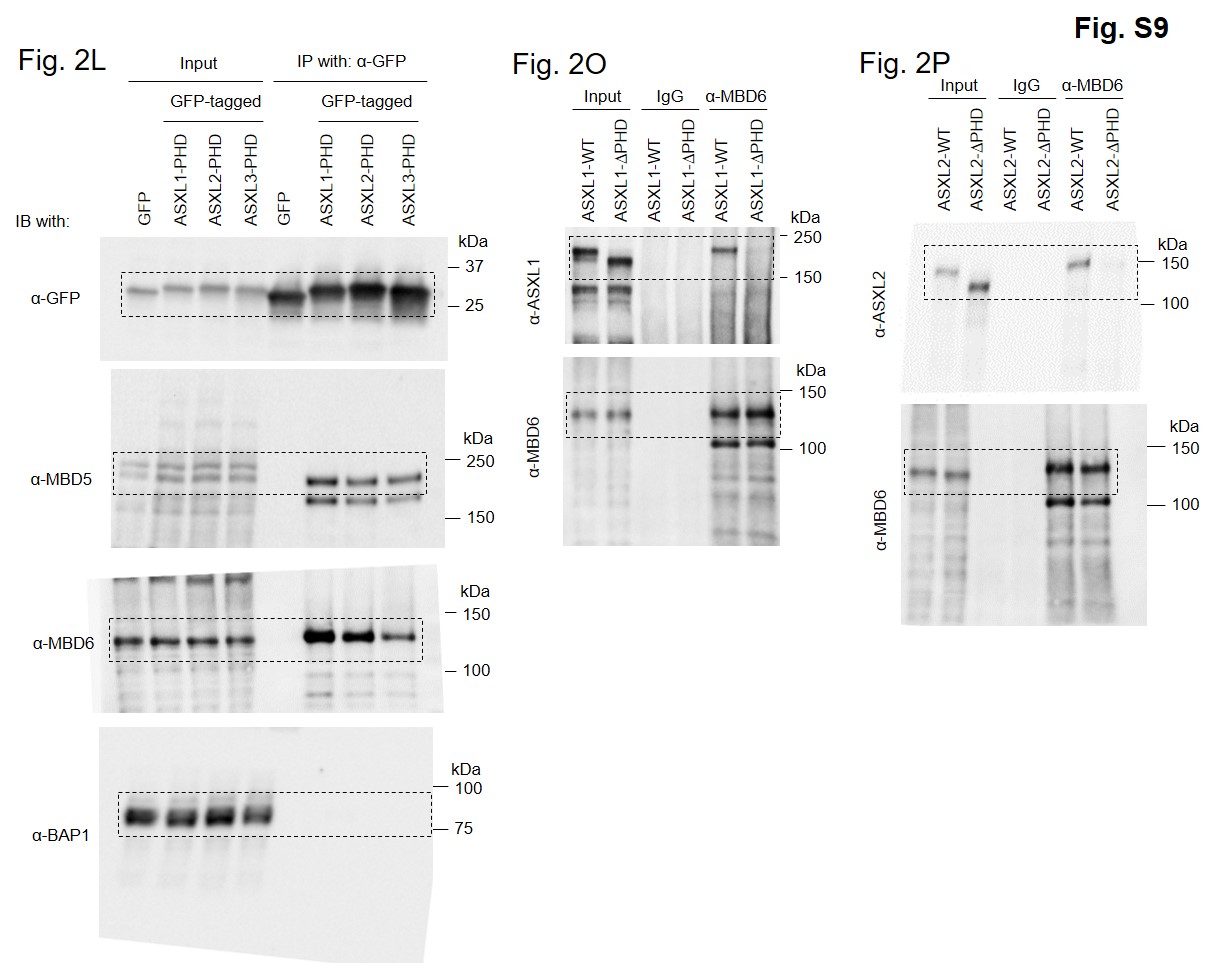
**

**Figure S9. Uncropped western blot gel images in Figure 2L-P.** The dotted line boxes highlight lanes used in figures.

**
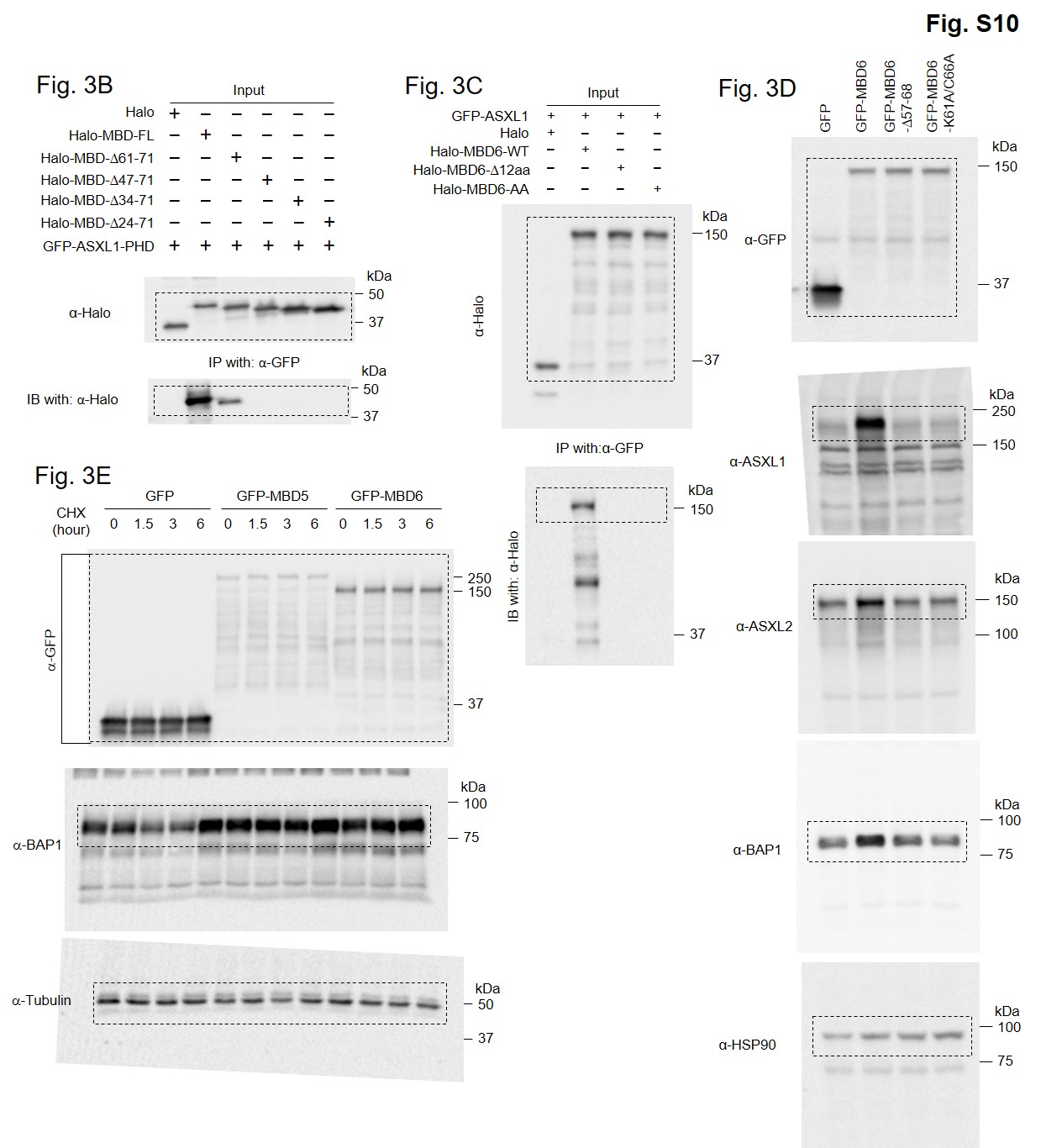
**

**Figure S10. Uncropped western blot gel images in Figure 3B-E.** The dotted line boxes highlight lanes used in figures.


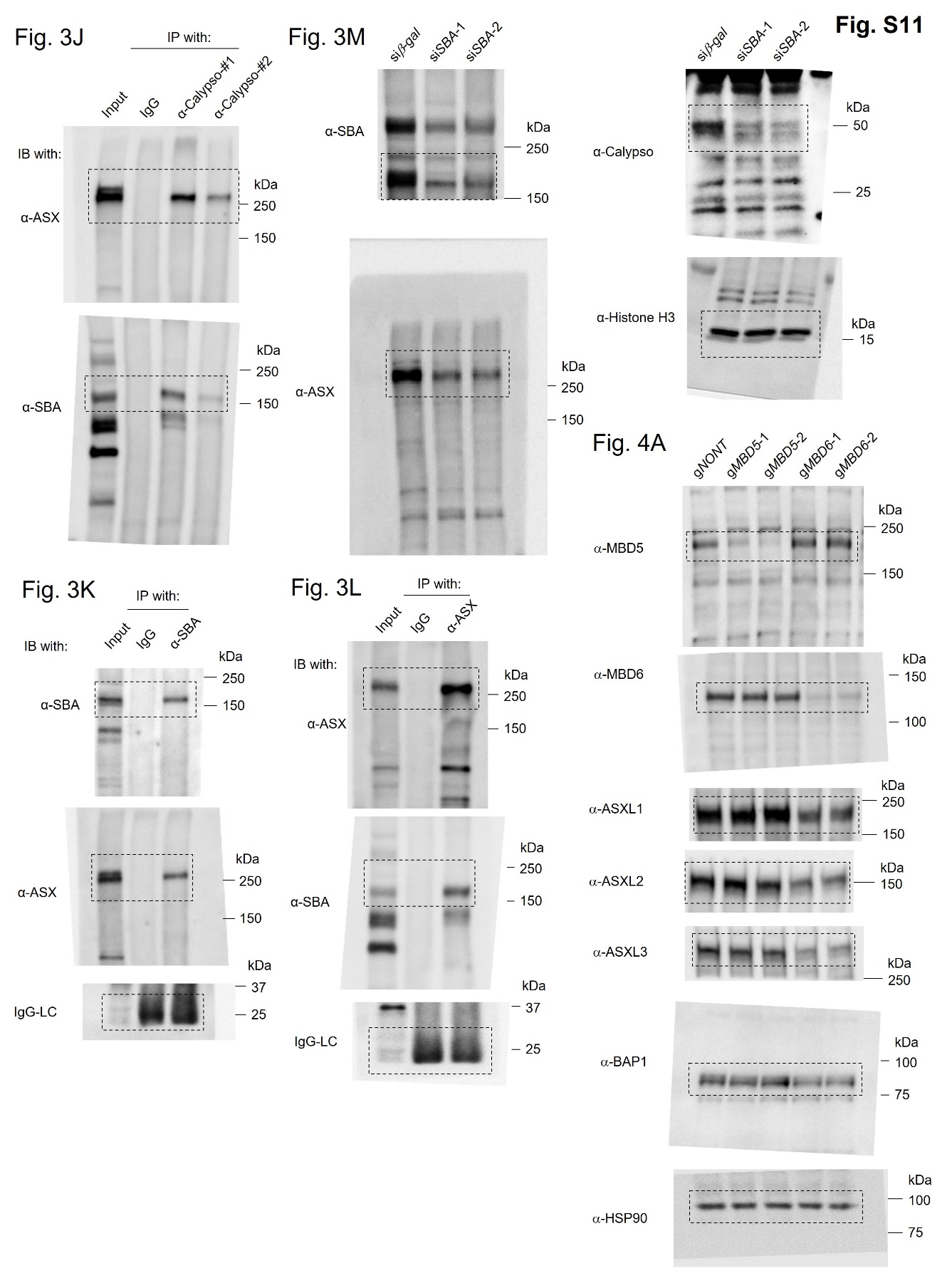


**Figure S11. Uncropped western blot gel images in Figure 3J-M and 4A.** The dotted line boxes highlight lanes used in figures.

**
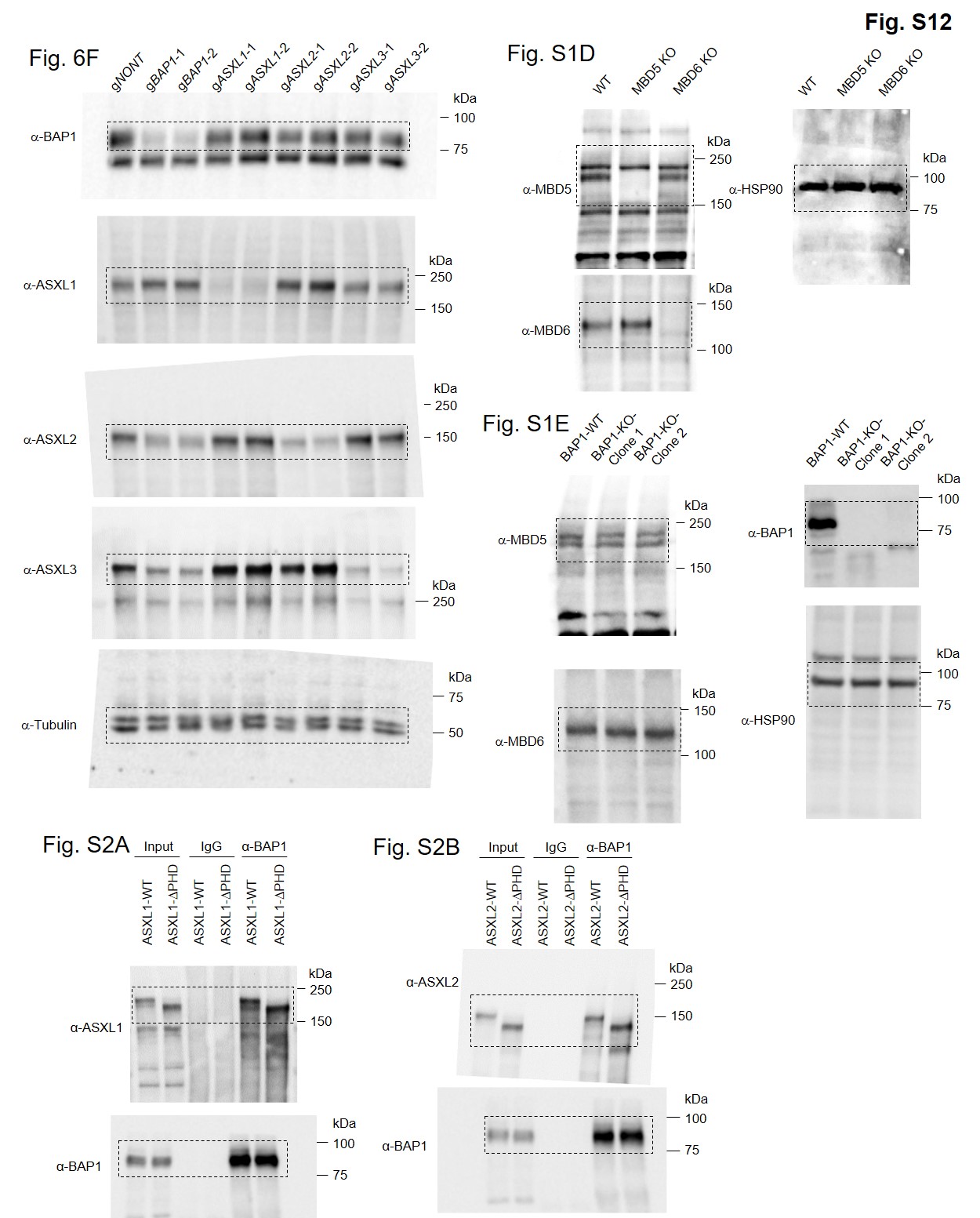
**

**Figure S12. Uncropped western blot gel images in Figure 6F, S1D-E, and S2A-B.** The dotted line boxes highlight lanes used in figures.

**
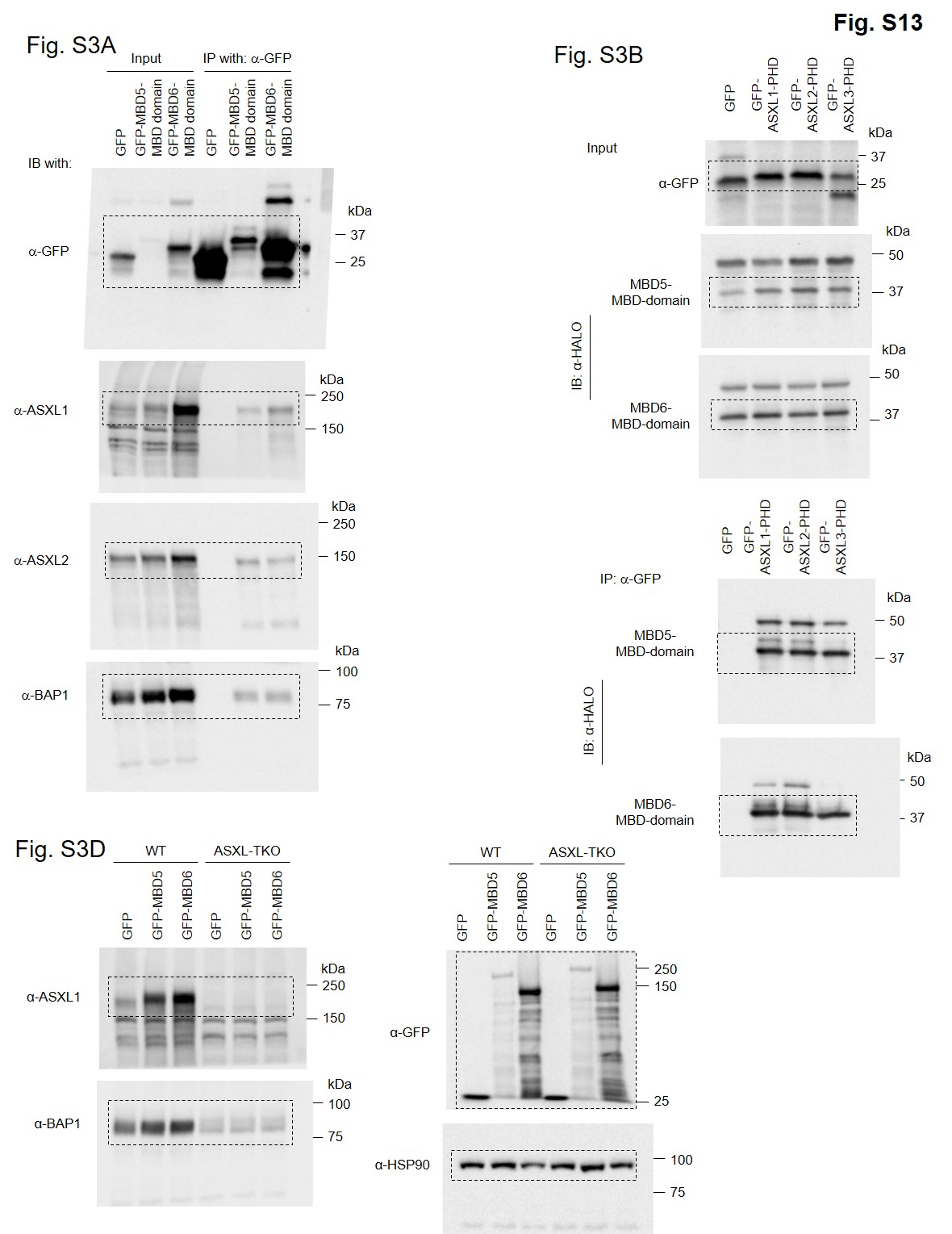
Figure S13. Uncropped western blot gel images in Figure S3A-B, and S3D.** The dotted line boxes highlight lanes used in figures.


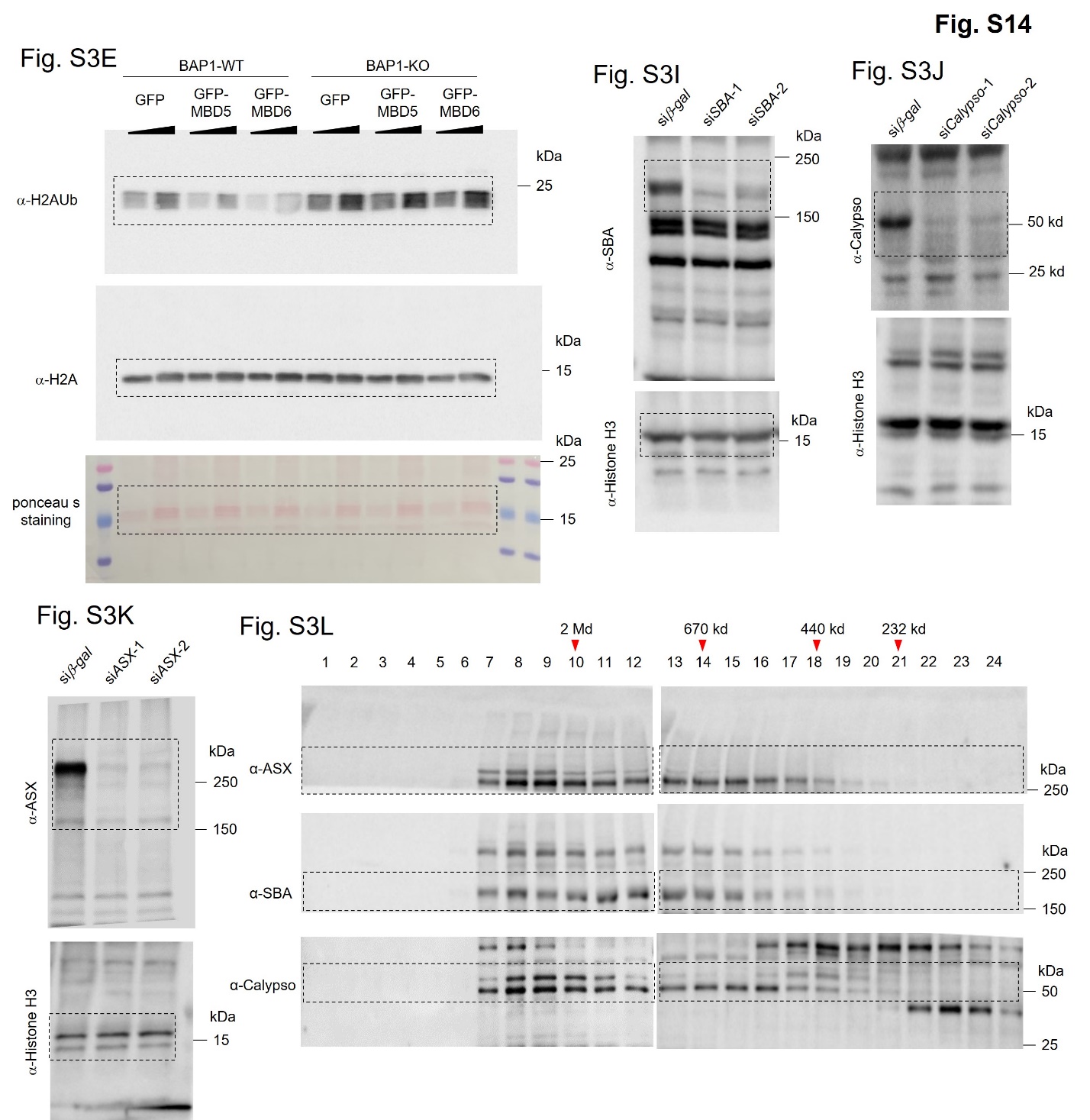


**Figure S14. Uncropped western blot gel images in Figure S3E and S3I-L.** The dotted line boxes highlight lanes used in figures.

**
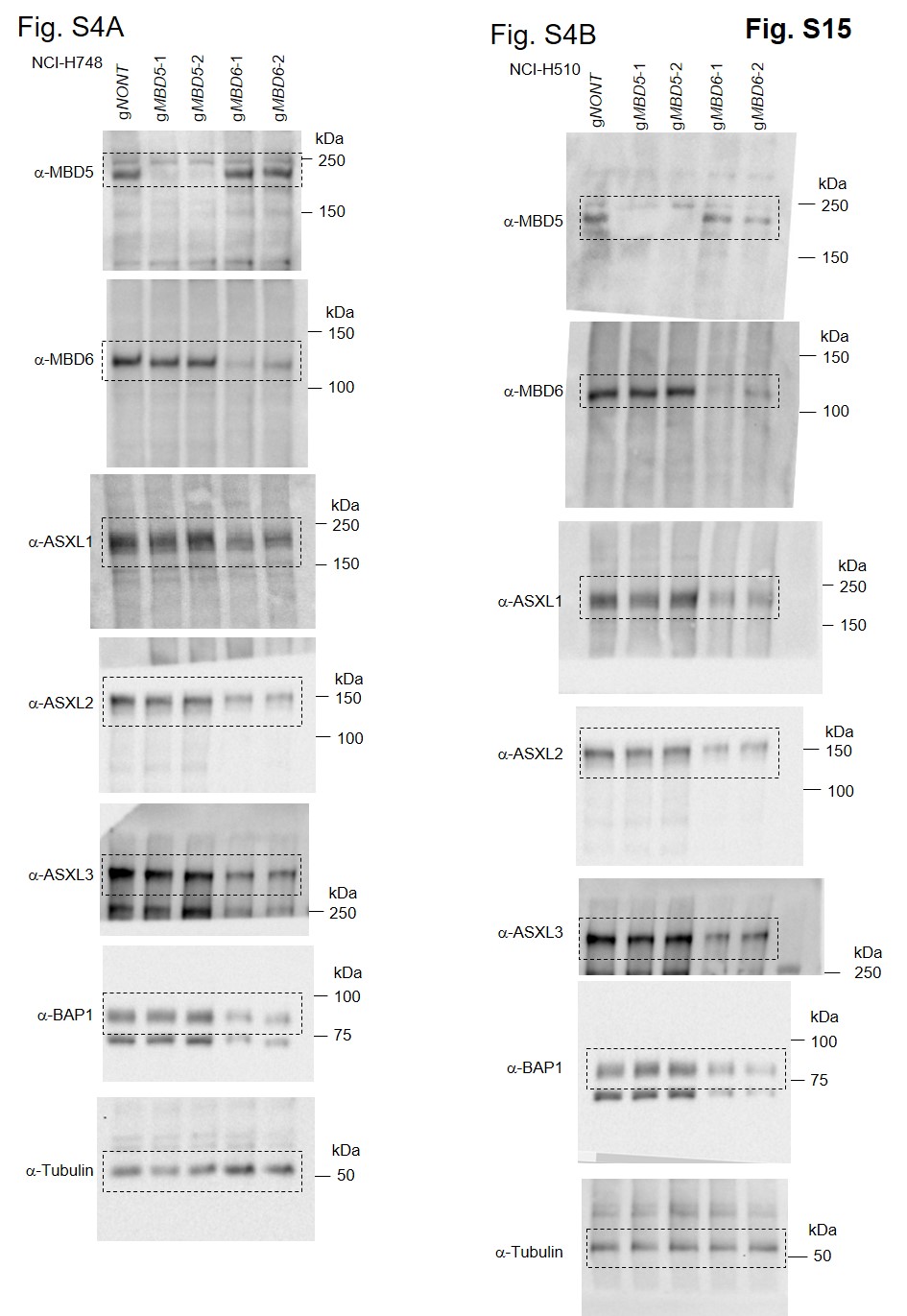
**

**Figure S15. Uncropped western blot gel images in Figure S4A and B.** The dotted line boxes highlight lanes used in figures.

**
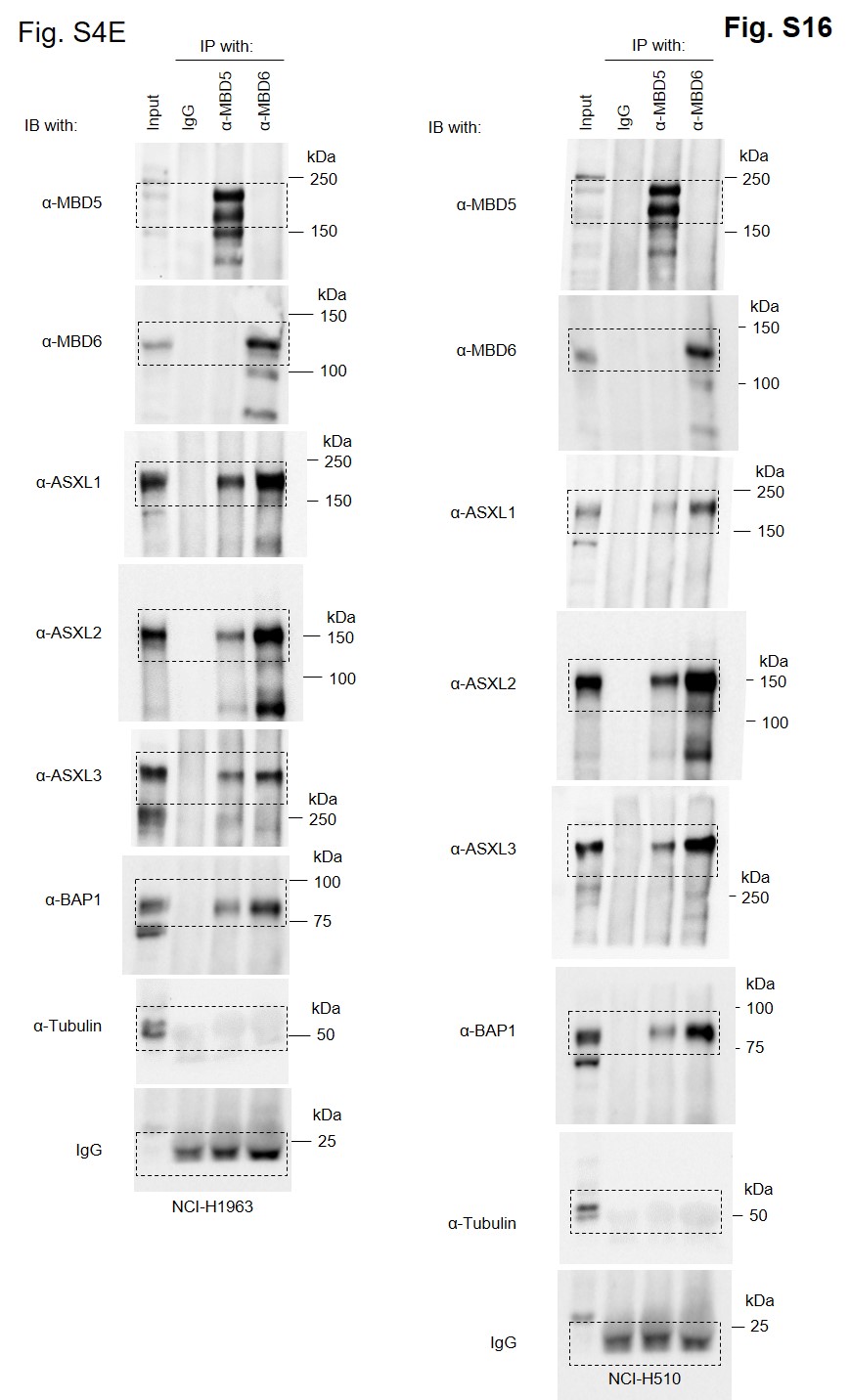
**

**Figure S16. Uncropped western blot gel images in Figure S4E.** The dotted line boxes highlight lanes used in figures.


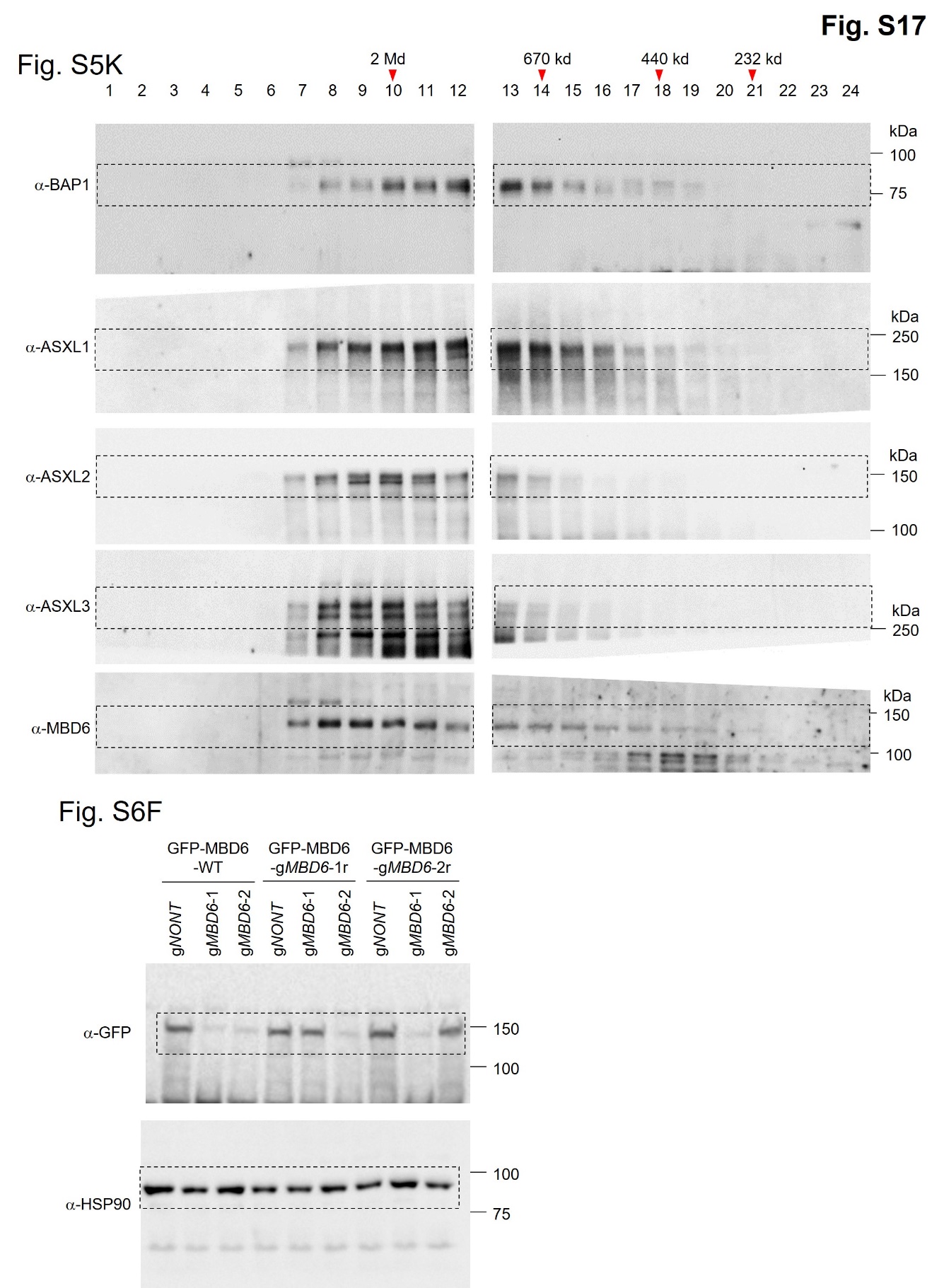


**Figure S17. Uncropped western blot gel images in Figure S5K and S6F.** The dotted line boxes highlight lanes used in figures.
