# Supplementary material for: MBD5 and MBD6 stabilize the BAP1 complex and promote BAP1-dependent cancer
Source: Genome Biol. 2022 Sep 30;23:206. doi: 10.1186/s13059-022-02776-x (PMC9523997; doi:10.1186/s13059-022-02776-x)
Supplement: Supplementary file 6 — Additional file 6. Review history. [file 13059_2022_2776_MOESM6_ESM.docx]

Review History

**First round of review**

**Reviewer 1**

**Were you able to assess all statistics in the manuscript, including the appropriateness of statistical tests used?**

No, I do not feel adequately qualified to assess the statistics.

**Comments to author:**

In this study, the authors studied the role of MBD5/6 in the BAP1 complex and showed that MBD5/6 indirectly interacts with BAP1 through ASXL and regulates BAP1 protein stability and function in gene transcription. They also showed that MBD6 is critical for small cell lung cancer (SCLC) growth using xenograft models.

Overall, this study conducted quite some transcriptomic and ChIP-seq analyses and detailed protein complex characterization, and most of the data are solid. Since MBD5/6 proteins have not been characterized in detail in previous studies, the findings also shed novel insights on our understanding of the BAP1 complex. The topic of the study certainly fits well with the scope of Genome Biology and should be of interest to the broad readership of the journal. The manuscript is also written well. The authors should address a few technical questions to further improve their manuscript.

In Fig. 3, the authors spent quite some efforts to characterize the interaction between MBD5/6 and ASXL/BAP1, and identified K61A/C66A mutant of MBD6 as the non-binding mutant. It is a pity that this mutant was never used in the subsequent functional studies of MBD6. This mutant will be important to address the question of whether MBD6's function in regulating gene expression and SCLC proliferation and tumor growth is at least partly dependent on its interaction with ASXL and BAP1. Specifically, they should re-express MBD6 WT and K61A/C66A mutant in MBD6 KO SCLC cells, and then examine whether mutant restoration rescues phenotypes caused by MBD6 deletion (WT restoration as a control), including cell proliferation (Fig. 4B), gene transcription (it would be nice to demonstrate this at the genome level by RNA-seq as shown in Fig. 4D, but minimally they should check a few selected genes as shown in Fig. 4G), and tumor growth (Fig. 4I-J).

Fig. 2B: here they need to show MBD5/6 blots for both inputs and IP (does ASXL1/2/3 KO depletes MBD6? It seems that this information was never mentioned in the paper. If there is no MBD5/6 protein in ASXL KO cells, then of course there would be no BAP1 in MBD IP).

They generated MBD5 or 6 KO cells in three SCLC cell lines (Fig. 4B), but only analyzed relevant protein levels in one cell line (Fig. 4A). Please also repeat the same IB analyses for the other two cell lines.

**Reviewer 2**

**Were you able to assess all statistics in the manuscript, including the appropriateness of statistical tests used?**

Yes, and I have assessed the statistics in my report.

**Comments to author:**

The Authors show that MBD5 and 6, which were previously shown to be part of BAP1-DUB-PR complex, are part of the latter through indirect interaction via binding to ASXL proteins. This binding is independent of BAP1 and is necessary for the interaction between MDB proteins and BAP1. Size exclusion chromatography of nuclear extracts suggests that BAP1 forms two different complexes with MBD5 and MBD6.

In HEK293 cells ChIP peaks enriched for MBD5/6 and BAP1 correspond to RNA related processes including RNA splicing, while peaks enriched for MBD6 and BAP1 are enriched for Ephrin signaling. Peaks enriched for MBD5 are poorly enriched for BAP1 occupancy. In SCLC cells MBD6 and BAP1 peaks are enriched for active chromatin markers and gene ontology shows similar pathways and processes related to neurogenesis.

A 12 amino acids stretch at the end of the MBD is necessary in MBD proteins to interact with PHD finger domain in ASXL C-terminus. The Authors identify in MBD6 two aa, K61 and C66, which are essential for interaction. Overexpression of MDB6 full length but not MBD6 with the deficient stretch of 12 a.a. results in higher basal levels of BAP1 and ASXL, and the same is observed for the Drosophila homologue of BAP1, Calypso and ASX by overexpression of the Drosophila homologue of MBD6, Sba.

Depletion of MBD6 in NCI-H1963 small lung cancer cell line results in decreased levels of BAP1 and ASXL proteins and a wide change of RNA expression where GO analysis reveals differentiation programs associated to cell of origin of SCLC. Depletion of MBD6 in H510 small lung cancer cell line results in decreased tumorigenesis.

The manuscript brings additional knowledge on the complex role of BAP1 on gene expression. However, several issues need to be addressed before it can be considered for publication defending the concept that MBD5 and 6 promote BAP1-dependent cancers.

Major:

-The finding that MBD5/6 are part of BAP1-DUB-PR through the interaction with ASXL proteins is not as surprising contrarily to what the Authors state since ASXL, but not BAP1, were previously shown to interact (Campagne et al Nat Commun 2019 Jan 21;10(1):348.doi: 10.1038/s41467-018-08255-x). Therefore, the Authors may change some statements accordingly.

-Some experimental procedures are not well described or not described at all: E.g. Plasmid generation is not described at all. The Authors use stably expressed GFP-tagged MBD5 and 6 in HEK293T cells, presumably N-terminal GFP, like in Baymaz et al 2014 (reference cited by the Authors)?

-Figure 1D: it would be an asset if the Authors could add some negative control such as Ezh2 in the co-IP experiments of MBD5 and 6 where BAP1 and ASXL are bound to show the specificity.

- The Authors generated anti MDB5/6 antibodies and in KO lines other bands are visible, especially for MBD5, where the contaminant band is more abundant compared to MBD5 band. Therefore, caution should be used when interpreting the data obtained through using these antibodies or appropriate controls such as the use of the KO cells should be used, e.g. in ChIP experiments.

- Would heatmaps of ChIP-seq data in Fig 1H change in ASXL ko ?

-Co-IP: in their previous study (Szczepanski et al Genome Med 2020) immunoprecipitation of ASXL proteins did not result in MBD5/6 pull-down in HEK293 cells. Can the Authors provide an explanation for the difference?

-In the size exclusion chromatography of nuclear extracts where BAP1 forms seems to form two different complexes with MBD5 and MBD6 and MBD5 is mostly as present in a high MW peak similar to what the Authors have previously shown (Szczepanski et al Genome Med 2020) of BAP1 complexes with BRD4 and ASXL3. However, the difference in fraction in MBD5 detection between fraction 12 and 13 seems very abrupt compared to the elution of the other 2 proteins where a gradient is detected. Can the Authors explain? Would these MBD5 and 6 peaks disappear in the absence of BAP1?

-Since MBD6 is involved in DNA damage response (DDR), and it is known that basal levels of DDR activation are present in cancer cells due to oncogenic-induced replicative stress. Therefore it plausible that MBD6 activation upon DDR would act as BAP1 stabilizer. However this mechanism should be further explored.

-Can the effects of MBD6 depletion in SCLC, e.g. tumorigenesis, be reversed by BAP1 and ASXL expression? In other words: while it makes sense that some transcriptional processes are different in the presence or absence of BAP1, is this sufficient for tumorigenesis? There might be BAP1-independent effects.

Minor:

- The Authors use stably expressed GFP-tagged MBD5 and should specify which isoform

- Methods: in IP description the Authors state that " IP experiments was performed as described before" but reference is missing.

-Figure 2A: What is the reason for lower ASXL2 in BAP1 ko cells?

**Authors Response**

**Point-by-point responses to the reviewers’ comments:**

Reviewer #1:

In this study, the authors studied the role of MBD5/6 in the BAP1 complex and showed that MBD5/6 indirectly interacts with BAP1 through ASXL and regulates BAP1 protein stability and function in gene transcription. They also showed that MBD6 is critical for small cell lung cancer (SCLC) growth using xenograft models.

Overall, this study conducted quite some transcriptomic and ChIP-seq analyses and detailed protein complex characterization, and most of the data are solid. Since MBD5/6 proteins have not been characterized in detail in previous studies, the findings also shed novel insights on our understanding of the BAP1 complex. The topic of the study certainly fits well with the scope of Genome Biology and should be of interest to the broad readership of the journal. The manuscript is also written well. The authors should address a few technical questions to further improve their manuscript.

In Fig. 3, the authors spent quite some efforts to characterize the interaction between MBD5/6 and ASXL/BAP1, and identified K61A/C66A mutant of MBD6 as the non-binding mutant. It is a pity that this mutant was never used in the subsequent functional studies of MBD6. This mutant will be important to address the question of whether MBD6's function in regulating gene expression and SCLC proliferation and tumor growth is at least partly dependent on its interaction with ASXL and BAP1. Specifically, they should re-express MBD6 WT and K61A/C66A mutant in MBD6 KO SCLC cells, and then examine whether mutant restoration rescues phenotypes caused by MBD6 deletion (WT restoration as a control), including cell proliferation (Fig. 4B), gene transcription (it would be nice to demonstrate this at the genome level by RNA-seq as shown in Fig. 4D, but minimally they should check a few selected genes as shown in Fig. 4G), and tumor growth (Fig. 4I-J).

*Reply: We thank the reviewer for pointing this out. Following the reviewer’s suggestion, we first generated CRISPR resistant MBD6 expressing plasmids by site-direct mutagenesis (Fig. S6E). After confirming that the mutations are resistant to MBD6-specific CRISPR sgRNA, we infected SCLC NCI-H510 cells with either GFP or GFP-tagged CRISPR resistant wild-type MBD6, or GFP-tagged CRISPR resistant K61A/C66A mutant MBD6 (Fig. S6F). As shown in Fig. S6G and H, we found that ectopically expressing wild-type but not mutant MBD6 at least partially rescued the cell growth rate and gene expression levels.*

Fig. 2B: here they need to show MBD5/6 blots for both inputs and IP (does ASXL1/2/3 KO depletes MBD6? It seems that this information was never mentioned in the paper. If there is no MBD5/6 protein in ASXL KO cells, then of course there would be no BAP1 in MBD IP).

*Reply: We thank the reviewer making this observation. Following the reviewer’s suggestion, we conducted immunoprecipitation experiments in ASXL WT/TKO cells with MBD5/6 antibodies and detected MBD5/6 protein levels in both inputs and IP samples. As shown in Figure 2B of the revised manuscript, we found that depletion of ASXL does not significantly reduce MBD5/6 levels.*

They generated MBD5 or 6 KO cells in three SCLC cell lines (Fig. 4B), but only analyzed relevant protein levels in one cell line (Fig. 4A). Please also repeat the same IB analyses for the other two cell lines.

*Reply: We thank the reviewer for this excellent point. Following the reviewer’s suggestion, we repeated the same experiments in the other two SCLC cell lines, NCI-H748 and NCI-H510. As shown in Figure S4A and S4B, depletion of MBD6 but not MBD5 also lead to a reduction of protein levels of BAP1 complex in both cell lines.*

Reviewer #2:

The Authors show that MBD5 and 6, which were previously shown to be part of BAP1-DUB-PR complex, are part of the latter through indirect interaction via binding to ASXL proteins. This binding is independent of BAP1 and is necessary for the interaction between MDB proteins and BAP1. Size exclusion chromatography of nuclear extracts suggests that BAP1 forms two different complexes with MBD5 and MBD6.

In HEK293 cells ChIP peaks enriched for MBD5/6 and BAP1 correspond to RNA related processes including RNA splicing, while peaks enriched for MBD6 and BAP1 are enriched for Ephrin signaling. Peaks enriched for MBD5 are poorly enriched for BAP1 occupancy. In SCLC cells MBD6 and BAP1 peaks are enriched for active chromatin markers and gene ontology shows similar pathways and processes related to neurogenesis.

A 12 amino acids stretch at the end of the MBD is necessary in MBD proteins to interact with PHD finger domain in ASXL C-terminus. The Authors identify in MBD6 two aa, K61 and C66, which are essential for interaction. Overexpression of MDB6 full length but not MBD6 with the deficient stretch of 12 a.a. results in higher basal levels of BAP1 and ASXL, and the same is observed for the Drosophila homologue of BAP1, Calypso and ASX by overexpression of the Drosophila homologue of MBD6, Sba.

Depletion of MBD6 in NCI-H1963 small lung cancer cell line results in decreased levels of BAP1 and ASXL proteins and a wide change of RNA expression where GO analysis reveals differentiation programs associated to cell of origin of SCLC. Depletion of MBD6 in H510 small lung cancer cell line results in decreased tumorigenesis.

The manuscript brings additional knowledge on the complex role of BAP1 on gene expression. However, several issues need to be addressed before it can be considered for publication defending the concept that MBD5 and 6 promote BAP1-dependent cancers.

Major:

-The finding that MBD5/6 are part of BAP1-DUB-PR through the interaction with ASXL proteins is not as surprising contrarily to what the Authors state since ASXL, but not BAP1, were previously shown to interact (Campagne et al Nat Commun 2019 Jan 21;10(1):348.doi: 10.1038/s41467-018-08255-x). Therefore, the Authors may change some statements accordingly.

*Reply: We thank the reviewer for pointing this out. We have included the paper as Ref 21 in the revised manuscript.*

-Some experimental procedures are not well described or not described at all: E.g. Plasmid generation is not described at all. The Authors use stably expressed GFP-tagged MBD5 and 6 in HEK293T cells, presumably N-terminal GFP, like in Baymaz et al 2014 (reference cited by the Authors)?

*Reply: We thank the reviewer for this excellent point. We have provided the plasmid information in the method section, and all the primers that were used to make the constructs in Supplementary Table 4.*

-Figure 1D: it would be an asset if the Authors could add some negative control such as Ezh2 in the co-IP experiments of MBD5 and 6 where BAP1 and ASXL are bound to show the specificity.

*Reply: We thank the reviewer for this excellent point. Following the reviewer’s suggestion, we have conducted IP experiment with MBD5/6-specific antibodies and determined the EZH2 levels in the immunoprecipitates. As shown in Figure 1D of the revised manuscript, there is no detectable interaction between EZH2 and MBD5/6 in HEK293T cells.*

- The Authors generated anti MDB5/6 antibodies and in KO lines other bands are visible, especially for MBD5, where the contaminant band is more abundant compared to MBD5 band. Therefore, caution should be used when interpreting the data obtained through using these antibodies or appropriate controls such as the use of the KO cells should be used, e.g. in ChIP experiments.

*Reply: We thank the reviewer for this excellent point. Following the reviewer’s suggestion, we validated the specificity of our antibodies in wild-type, MBD5-KO, and MBD6-KO cells. As shown in Figure S1F of the revised manuscript, we found that a vast majority of these MBD5/6 peaks are gone in the KO cells.*

- Would heatmaps of ChIP-seq data in Fig 1H change in ASXL ko?

*Reply: We thank the reviewer for this excellent point. Following the reviewer’s suggestion, we conducted ChIP-seq to determine chromatin occupancy of BAP1/MBD5/MBD6 in ASXL-WT/TKO cells. As shown in Figure S2C, we found loss of ASXL reduced BAP1 occupancy but not MBD5/6 at BAP1 occupied regions, which is consistent with our western blot results shown in Figure 2B.*

-Co-IP: in their previous study (Szczepanski et al Genome Med 2020) immunoprecipitation of ASXL proteins did not result in MBD5/6 pull-down in HEK293 cells. Can the Authors provide an explanation for the difference?

*Reply: We thank the reviewer for this excellent point. Our previous results are very similar to that in the Nature paper published by Dr. Müller’s lab (Nature. volume 465, pages243–247 (2010)). During the protein purification, the C-terminal end of ASXL may be truncated (Fig. 1b of the above paper), and therefore the MBD5/6 (SBA in drosophila) might be hard to detect in the purification.*

-In the size exclusion chromatography of nuclear extracts where BAP1 forms seems to form two different complexes with MBD5 and MBD6 and MBD5 is mostly as present in a high MW peak similar to what the Authors have previously shown (Szczepanski et al Genome Med 2020) of BAP1 complexes with BRD4 and ASXL3. However, the difference in fraction in MBD5 detection between fraction 12 and 13 seems very abrupt compared to the elution of the other 2 proteins where a gradient is detected. Can the Authors explain? Would these MBD5 and 6 peaks disappear in the absence of BAP1?

*Reply: We thank the reviewer for this excellent point. Actually, the size exclusion results that contains both MBD5 and MBD6 are from HEK293T cells, which express very low levels of ASXL3. In our previous paper (Szczepanski et al Genome Med 2020), the size exclusion was performed in SCLC cell line NCI-H1963, in which the ASXL3 is highly expressed. In fact, in Figure S5K of our manuscript, we have shown the size exclusion data in NCI-H1963 cells. These results remain consistent with our previous publication (Szczepanski et al Genome Med 2020). Interestingly, we have noticed a significant shift of MBD6 to high molecular weight peak, which is probably due to the expression of ASXL3 (Fig. S5K) and ASXL3-associated factor(s), such as BRD4. For the MBD5 protein levels in the size exclusion experiment, we think this is a technical problem such as the quality of the homemade SDS page gel. We have repeated the experiment again and have shown the result in the revised manuscript. For the last question, we expect that MBD5/6 peaks may not disappear or reduce significantly in the absence of BAP1 because the remaining ASXL1-3 and their associated factors may be sufficient to form a complex with MBD5/6 (Fig. 2A).*

-Since MBD6 is involved in DNA damage response (DDR), and it is known that basal levels of DDR activation are present in cancer cells due to oncogenic-induced replicative stress. Therefore it plausible that MBD6 activation upon DDR would act as BAP1 stabilizer. However this mechanism should be further explored.

*Reply: We thank the reviewer for this excellent point. We agree with the reviewer that the basal levels of DNA damage response may induce activation of MBD6, and thereby stabilizing BAP1 protein in SCLC cells. Therefore, depletion of BAP1 or MBD6 may strongly induce DNA damage in these cells. We think this is a very exciting project and we would like to follow it up in our future studies. We hope the reviewer agrees with us that it is out of the scope of our current manuscript.*

-Can the effects of MBD6 depletion in SCLC, e.g. tumorigenesis, be reversed by BAP1 and ASXL expression? In other words: while it makes sense that some transcriptional processes are different in the presence or absence of BAP1, is this sufficient for tumorigenesis? There might be BAP1-independent effects.

*Reply: We thank the reviewer for this excellent point. It is evident that loss of MBD6 reduces all three ASXL proteins, which were required for the protein stability of BAP1 (Fig. 4A). Therefore, restoration of BAP1 may not rescue the phenotype of MBD6 depletion. However, it is possible that restoration of all three ASXLs (ASXL1-3) together may rescue or partially rescue the phenotype of MBD6 depletion. However, due to technical problems, it may be very difficult to express all three ASXLs (ASXL1: ~200 kd, ASXL2: ~150 kd, and ASXL3: ~300 kd) at proper levels in SCLC cell lines. We will try to develop a new strategy, such as establishing CRIPSRa in SCLC cell lines in our future studies.*

Minor:

- The Authors use stably expressed GFP-tagged MBD5 and should specify which isoform.

*Reply: We expressed the isoform 1 because this isoform has been chosen as the canonical sequence. Following the reviewer’s suggestion, we have included the information in the method section of the revised manuscript.*

- Methods: in IP description the Authors state that " IP experiments was performed as described before" but reference is missing.

*Reply: We thank the reviewer for pointing this out. We have added the corresponding reference in the revised manuscript.*

-Figure 2A: What is the reason for lower ASXL2 in BAP1 ko cells?

*Reply: We thank the reviewer for pointing this out. It has been reported that BAP1 maintains the mono-ubiquitin modification of ASXL2 in a catalytically-independent manner (Nature Communications. 2018 Oct 22; 9(1):4385, Figure 1e).*

**Second round of review**

**Reviewer 1**

The authors have addressed this reviewer's comments. The manuscript can be accepted for publication.

**Reviewer 2**

The Authors have revised the manuscript addressing all major concerns in an adequate manner.
